# The osteometric identification of castrated reindeer (*Rangifer tarandus*) and the significance of castration in tracing human-animal relationships in the North

**DOI:** 10.1007/s12520-022-01696-y

**Published:** 2022-12-09

**Authors:** Mathilde van den Berg, Henri Wallen, Anna-Kaisa Salmi

**Affiliations:** 1 Archaeology, History, Culture and Communication Studies, Faculty of Humanities, University of Oulu, Oulu, Finland; 2 Arctic Centre, University of Lapland, Rovaniemi, Finland

**Keywords:** Domestication, Zooarchaeology, Cervidae, Arctic, Reindeer herding, Human-reindeer relationships

## Abstract

Reindeer are the only domestic cervid and have formed the cosmologies and practical daily lives of numerous peoples in the Northern Hemisphere for thousands of years. The questions of when, how, and where reindeer domestication originated and how it developed remain one of the scientific enigmas of our time. The practice of reindeer castration is an essential feature of all communities practicing reindeer herding today. It has probably been one of the most important interventions in the reindeer’s life cycle and biology that marked the start of domesticating human-reindeer relationships long ago. Castration is and has been essential for reindeer taming, control, training, herd management, and ritual practices. Unsuitably, to this present day, there are no methods zooarchaeologists can employ to distinguish a reindeer gelding from a reindeer bull in the archaeological record. In this current paper, we outline a new method that presents the possibility of differentiating between full males, castrated males, and females based on osteometric features. We measured the leg bones and pelvis of the complete or partial skeletons of 97 adult modern domestic reindeer individuals to determine the precise effects castration has on skeletal size and morphology. We explored our osteometric dataset with different statistical methods. We found a clear separation of the two male groups in the radioulna, humerus, and femur but in the tibia and metapodials to a lesser extent. Osteometric depth and width were generally more affected than the longitudinal axis. Females were easily distinguishable from castrates and full males based on nearly every bone measurement. Our analysis shows that reindeer castration can be proven through osteometric analysis.

**Supplementary Information:**

The online version contains supplementary material available at 10.1007/s12520-022-01696-y.

## Introduction

One of the major questions in human-animal relationships is the origins and transformations of reindeer (*Rangifer tarandus* Linnaeus, 1758) breeding and herding systems by the many reindeer herding communities inhabiting the circumpolar North. Reindeer have been a source of livelihood and have shaped the cultures of these circumpolar communities for thousands of years. Initially as the most important game species, but later in Eurasia also as a source of labor (Gordon 2003; Ventsel 2006; Helskog 2011; Anderson et al. 2019), transforming the reindeer into a working companion and friend (Laufer 1917 p. 142; Vitebsky 2005 p. 95), and as a source of food and other subsistence resources, making numerous cultures across Eurasia physically and culturally dependent on this species (e.g., Kofinas et al. 2000; Huntington and Fox 2005). Nevertheless, despite reindeer domestication being such an essential part of the history of numerous cultures in the Northern hemisphere, its origin and development remain, although widely researched, still highly debated and controversial (e.g., Røed et al. 2011; Sommerseth 2011).

The reindeer was domesticated in Siberia and Fennoscandia, probably in separate domestication events (Røed et al. 2008; Melak et al. 2020). The earliest evidence of reindeer domestication comes from Siberia, in which artifacts related to training transport reindeer have been dated to the start of the Common Era (Losey et al. 2020). Nowadays, there is a broad acceptance of the emergence of reindeer herding In Fennoscandia during the second half of the Late Iron Age (800–1050 AD) (Aronsson 1991; Storli 1994, pp 64–70; Bergman et al. 2008; Hedman et al. 2015). This region’s initial domestication of reindeer likely began much earlier than the Late Iron Age however, as hunter-gatherers used reindeer as decoys in wild reindeer hunting and for transport purposes (Hansen and Olsen 2014).

Regardless of the ongoing research, the knowledge and understanding of the place, timing, and nature of the varied reindeer management strategies of the past remain obscure. There is a myriad of reasons for this, including geographical and temporal variations of types of reindeer herding (e.g., Lundmark 2007; Andersen 2011, p 11; Sommerseth 2011), supplementary livelihoods (Tegengren 1952; Hultblad 1968, p 206; Nielssen 1986; Lundmark 1982, p 144; Lundmark 2007), and the elusiveness of the archaeology of (semi-)nomadic peoples which largely stems from its archaeological invisibility (Seitsonen et al. 2018; Tervaniemi and Magga 2019; Seitsonen 2020). The difficulty in interpreting reindeer bone finds from archaeological sites is that domestic reindeer lack clear features of the domestication syndrome and the phenotypic plasticity of the reindeer skeleton. What further muddles the issue is that the ecotypes present in both Fennoscandia and Siberia are phenotypically very similar. For example, the two ecotypes present in Fennoscandia (*Rangifer tarandus fennicus* and *Rangifer tarandus tarandus,* which includes domestic reindeer) overlap in size and have similar osteo-morphologies (Nieminen and Helle 1980; Grøn 2011; Puputti and Niskanen 2009; Salmi and Heino 2019; Pelletier et al. 2020).

An integral and inseparable aspect of all reindeer herding communities of the circumpolar North is reindeer castration. It is of considerable significance for reindeer training, taming, control, and for the reindeer herding strategies of today’s herding cultures (Acerbi 1802; Hatt 1918; Wiklund 1918, p 256, p 271; Mirov 1945; Rönnow 1949; Wustmann 1951; Skjenneberg and Slagsvold 1979, pp 278–283; Vainshtein 1980, p 126; Beach 1981, p 129, p 203; Ingold 1986; Svanberg and Lindin 1986 pp 161–162; Paine 1994; Etylin 2007; Vitebsky 2005, p 44, pp 94–95, pp 136–137, p 279; Stammler 2005, p 57, p 171; Stépanoff 2012; Bjørklund 2013; Ragagnin 2017a, b; Salmi et al. 2020b), as well as for ritual purposes (Vitebsky 2005, p 279). Several authors argue that before any kind of herds became established in Siberia and Fennoscandia, reindeer domestication started with the taming of castrated males for transportation purposes (Ingold 1986; Bjørklund 2013, p 177). This makes it one of the most important elements to consider in questions relating to the origin, spread, and development of domestic human-reindeer relationships. Although the importance of castrates in the past and present reindeer cultures is widely recognized, no methods exist that can discern a reindeer gelding from a reindeer bull. Besides, no studies have yet addressed the significance of reindeer castration in light of the possibilities of its detection in archaeological assemblages to this day.

One effective approach for documenting castrated ungulates is with osteometric and osteomorphological analyses. This method has been performed on, for example, sheep (Davis 2000; Popkin et al. 2012) and has shown the most promise for long bones. The premise of this method is that bone growth is linked to epiphyseal fusion (Silver 1963; Kennedy et al. 1999). Several studied species clearly show altered patterns of epiphyseal fusion for the castrates relative to both males and females (Hatting 1983; Noddle 1974; Moran and O’Connor 1994; Davis 2000), which allows for changes in the osteological development of the long bones (Hobday 1914; Silberberg and Silberberg 1971; Davis 2000; Popkin et al. 2012) and can thus be detected through osteometrics and osteomorphometrics.

This study presents new methods to discern castrated from full male and female domestic reindeer (*Rangifer tarandus tarandus*) based on postcranial skeletal measurements. We explore various statistical analyses and simple variable combinations to differentiate the castrates from the other two groups in terms of bone size and shape. This study is the first of its kind to evaluate a new method to detect castration in reindeer bones. The future use of our method on fossil reindeer bone assemblages could aid in the evaluation of the use and cultural context of the prevalence of castrated reindeer through time, shed new light on the origins and development of the many reindeer herding cultures today, and hints at the possibility of tracing domestication through the identification of castration for other mammal species besides reindeer.

## Background

### Reindeer castration and herd management

The castration of reindeer is an age-old practice and is of great importance for past and present reindeer herding societies. Castrated reindeer are used and were used as working animals, as a meat source, for herd management strategies, and for ritual practices. Currently, castrated reindeer are also used as tourist and racing reindeer. The first years of a reindeer’s life are the most important, as it is during that time that the herder decides the purpose of each individual in the herd structure and economic management. The strategic planning of the function of a reindeer individual will be based on its age, sex, and individual qualities (Magga 2006).

The importance of castrated reindeer is reflected in the extensive vocabulary that reindeer herding people hold to these categories of reindeer, as well as common expressions used among reindeer herding peoples. An extensive terminology is devoted to the different qualities, age classes, work capacity, and behavior traits of working deer among herding cultures in Fennoscandia and Siberia (Paine 1994, p 80; Magga 2006; Ragagnin 2012; Ragagnin 2017a; Ragagnin 2017b).

The status of castrated reindeer in, e.g., Sámi society is illustrated by the expressions that Acerbi came across during his travels through Lapland in the years 1798 and 1799. The expressions clearly articulate the value of these animals and their significance among reindeer owners. For example, Acerbi mentions that he had heard somebody in an elevated and boasting mood exclaim, “*heerge zhiouga*” or “*I am a castrated reindeer*.” Another expression is “*uartzejetz*,” or “*they are absolutely a castrated reindeer*,” which is used when somebody is eligible for the highest form of praise (Acerbi 1802, p 149). He mentions that it is not uncommon that anything of value is said to be worth a gelded reindeer. If a reindeer herder wants to compliment another herder, he can tell him that he appreciates him as much as a gelded reindeer (Acerbi 1802, p 200).

If a reindeer is castrated to become a *haergi* (trained castrated reindeer in the North Sámi language), herders look forward to several reindeer features after castration. Castrated bulls have calmer personalities and are easier to train than full males (Skjenneberg and Slagsvold 1979, p 278). They avoid hormonal shifts and general exhaustion during the rut and, consequently, retain their weight better than full males (Beach 1981, p 129; Skjenneberg and Slagsvold 1979, p 278). This allows them to grow large and muscular (Aikio 1989) so that also, during the winter, they are fit to work. In contrast, full males are more exhausted and emaciated during winter. There is always a chance that full males do not make it through the winter or spring because they have exhausted themselves during the rutting season (Paine 1994, pp 25–28). This makes castration a safer option if the herder has invested in training the animal (Van den Berg 2022, unpublished manuscript).

**Fig. 1:**
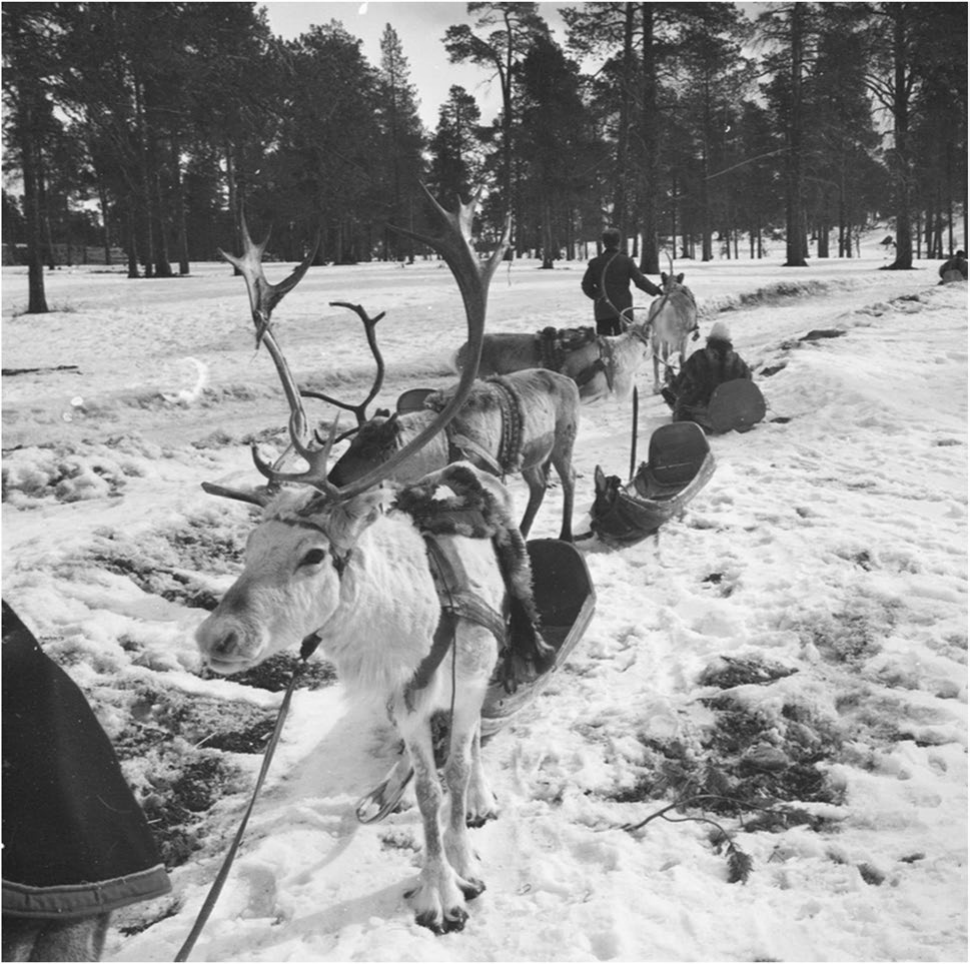
Castrated reindeer in a reindeer caravan (*rai’do* in Northern Sámi language) in Inari, 1938. Before the introduction of the snowmobile, castrated reindeer were used in reindeer caravans during migrations between pastures, villages, and trade points. (Photo: Aarne Pietinen Oy 1938, Finnish Heritage Agency 2021)

**Fig. 2:**
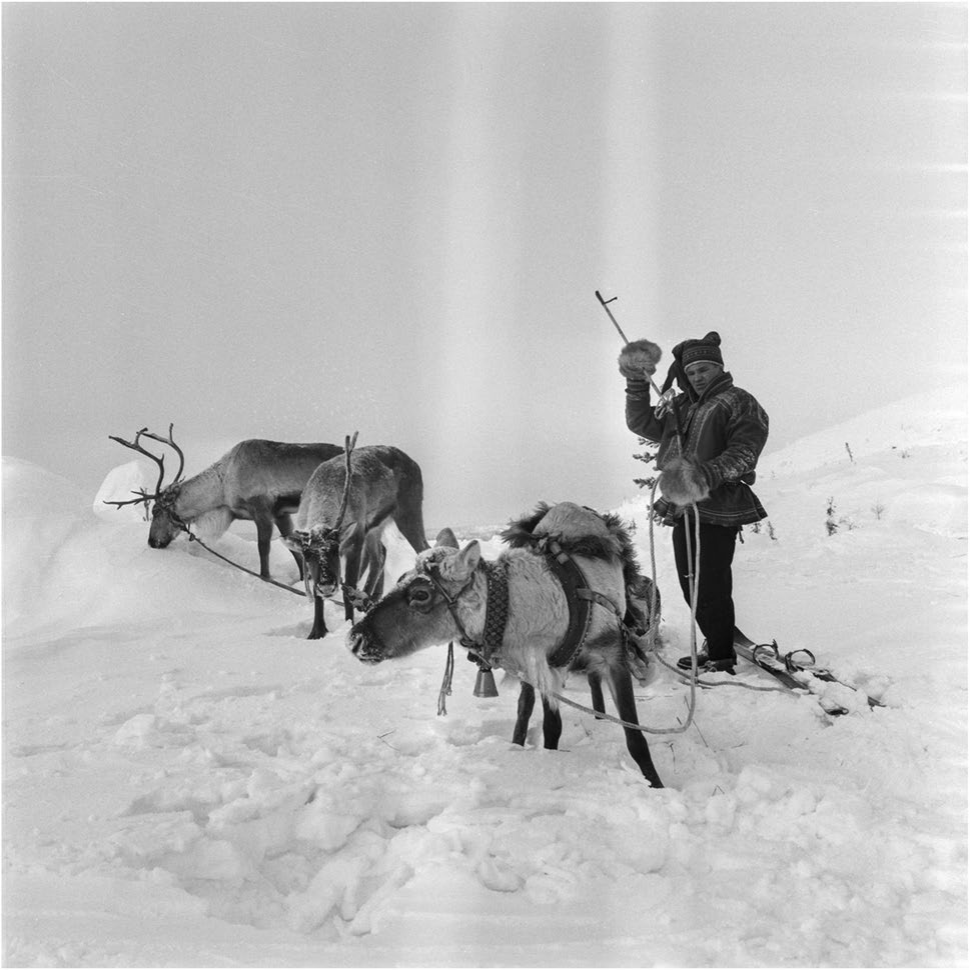
Castrated reindeer with a reindeer herder on skis, 1962. These reindeer were specially trained for transport and other purposes and offered mobility to every household member. Castrated reindeer retain their antlers in the winter, unlike full males (Photo: U.A. Saarinen 1962, Finnish Heritage Agency 2021)

Before the introduction of the snowmobile, transport reindeer (Figs. 1 and 2) were the most valuable portion of the reindeer herd. They were used to pull the sleds, carry the household supplies, were employed in the reindeer caravan, and were used as lead reindeer during migrations (e.g., Collinder 1949, p 95; Beach 1993, p 14). These valuable reindeer were kept close to the herders and guarded so they would not fall prey to predators (Beach 1981, pp 84–85). During the snowless part of the year, the working reindeer were employed as pack animals and, during the winter months, as draft reindeer in front of sleds (Pitkänen et al. 1984, p 55). Traditionally, castrated reindeer and sometimes sterile does were used as working animals (Collinder 1949, p 96). It is asserted by Acerbi (1802, p 202) that prosperous reindeer herders made use of castrated reindeer for drawing sleds, while less wealthy reindeer herders contented themselves with sleds pulled by female reindeer. The death of one of these castrated animals was a considerable blow for a reindeer herding family. Many families have been delayed in their bi-annual migrations due to the death of their working reindeer (Beach 1981, pp 84–85). It was not uncommon for all household members to have their own trained castrated reindeer, which were remarkably tame and had names (Aikio 1989). For example, Paine (1994, p 80) reported from the Finnmark Sámi in Norway during the 1950s that each of the teenage children had their own *haergi* to make unaccompanied trips to visit boyfriends and girlfriends in other camps.

In large-scale pastoralism, economically speaking, a reindeer herd consists of three types of reindeer: breeding reindeer, reindeer for meat, and castrated reindeer trained for special purposes. The breeding reindeer are the does and a selected number of sires, as only a few bucks are needed for breeding (one breeding bull could sire more than 10 does, sometimes up to 100 (Pitkänen et al. 1984, pp 93–94)). Most males are selected for slaughter or castration, of which many of the slaughtered ones will be castrated too (Collinder 1949, p 112; Paine 1994, p 218; Nilsen 1998). The meat of castrated reindeer is considered tender and fat (Paine 1994, pp 25–28), while the meat of full males is considered distasteful, especially during or right after the rut (Van den Berg 2022, unpublished manuscript). Besides, castrating a selection of the bulls of age enables the herders to steer the rut in a certain direction by retracting the males that are not seen as desirable to sire offspring (Paine 1994, pp 25–28).

Castrated reindeer in a herd setting provide several benefits to the herder from a behavioral perspective. They tend to stray less from the herd than bulls (Beach 1993, pp 71–73) and are calm animals who help the herd to keep gathered and stimulate the rest of the herd to quiet down (Etylin 2007). In this sense, they are a great promoter of herd centricity (Paine 1994, pp 25–28). They are also thought to lower the females’ general activity level, contributing to the herd’s increased net energy gain (Nilsen 1998). During the winter, castrates are crucial in the herd as they are strong enough to break through the ice cover and permit the smaller animals to graze. This is important during winters with a lot of climate variability that can raise difficult grazing conditions (Etylin 2007; Reinert et al. 2008).

### Age, methods, and strengths of castration

The notable thing about reindeer castration is that it gives the herder many options. The choice is not merely between “to castrate or not” but involves both strength and age, giving the herder a considerable number of different outcomes of the castrated animal tailored to the purpose of the reindeer the herder has in mind.

In Fennoscandia, working reindeer are usually castrated between 3 and 4 years of age (Paine 1994, pp 25–28; Nieminen and Pietilä 1999, p 122; Korhonen 2008, pp 132–133; Salmi and Niinimäki 2016). The reindeer selected to become working animals are and used to be good-natured and strong (Pitkänen et al. 1984, p 55, p 93; Paine 1994, pp 25–28). It is thought that if castrated too early, the reindeer develops poorly with feminine features and light forequarters (Rönnow 1949; Skjenneberg and Slagsvold 1979, p 283). Therefore, in current and historical Fennoscandia, reindeer are castrated and were usually not castrated before they reached near maturity. Not all animals selected as working animals make it through the “selection phase.” Some animals might prove challenging to train or otherwise unfit for the work or purpose the herder had in mind. These animals, although castrated, would either be consumed directly or let free in the herd to roam until the herder chooses to consume them (Paine 1994, pp 26–28, p 80).

Other (historical) accounts of reindeer castration among the Sámi reindeer herders have suggested that earlier ages of castration were also in practice. For example, Linnaeus observed in his journey through Lapland that animals could be castrated at 2.5 years of age (Carl Linnaeus 1732, in Graves 1995). Rönnow noticed among poor Sami herders in Jokkmokk parish, Sweden, that even 2-year-olds could be castrated when there were insufficient meat reindeer available for slaughter (Rönnow 1949).

Animals from different age classes might be selected for castration with the prospect of their slaughter later in the year for either domestic consumption or sale or both (Paine 1994, pp 25–28). Bulls seen unfit for breeding duties are castrated likewise (Pitkänen et al. 1984, p 55). Part of the 3-year-old males can be castrated for tender and fat meat, while the rest of this age group can enter the rut. Some of the 4-year-old males are allowed to enter the rut too. Of this age group, the ones considered fit as working animals might be castrated early in the summer, while those castrated for meat purposes might be castrated later in the year (Paine 1994, pp 25–28). Today’s meat market in Fennoscandia focuses on calf meat, but before the 1980s, 5-year-old castrated bulls were slaughtered and sold for the meat market. The meat of these older bulls is considered fat, strong in flavor, and preferred by the Sámi herders who keep and slaughter these animals for domestic use (Van den Berg, 2022, unpublished manuscript). For herd management reasons, reindeer might be castrated up to 6 years of age (e.g., Rönnow 1949) as senior bulls might be castrated to reduce aggressive competition between bulls at the rut or if they are likely to succumb to fatal exhaustion during the following winter (Paine 1994, pp 25–28).

Since ancient times, Sámi reindeer herders have traditionally used their teeth to castrate reindeer (Acerbi 1802, p 200; Wiklund 1918, p 256, pp 271–272; Wustmann 1951; Bosi 1960, pp 110–114; Spencer 1978, p 46; Carl Linnaeus 1732, in Graves 1995). This same method has also been prevalent among reindeer herding cultures in Siberia and might be the most ancient castration technique (Vainsthein 1980, p 112; Svanberg and Lindin 1986, pp 161–162; Arzyutov and Liubliskaia 2018, p 81, pp 111–112; Arzyutov et al. 2021, pp 359–360). The glands were bitten, and the scrotum would be carefully massaged afterward (Rönnow 1949; Carl Linnaeus 1732 in Graves 1995). The advantages of this method are that the skin remains imperforated, which lowers the risk of infection, and testosterone production continues to some extent if a portion of the gland is deliberately left intact. The newly castrated reindeer gelding would appear unwell for a couple of days after the procedure (Rönnow 1949).

It is, therefore, quite logical that in Sámi vocabulary, the term for castration “gasket” comes from the verb “to bite” (Skjenneberg and Slagsvold 1979, p 279). Nowadays, castration through biting is no longer used in Fennoscandia. It was banned in Norway, Sweden, and Finland during the last half of the twentieth century (Paine 1994, pp 26–28). Castration is now to be done using specifically designed tongs, of which different varieties are in use, which crush the tissue in the spermatic cord (Skjenneberg and Slagsvold 1979, p 279; Beach 1981, p 129; Paine 1994, pp 26–28; Regjeringen.no 2000).

Different “strengths” of castration regulate the hormonal flow, traditionally depending on how much of the bitten gland would be left intact (Skjenneberg and Slagsvold 1979, p 279; Van den Berg 2022, unpublished manuscript). Different strengths of castration allow for different properties of the castrated animal. If done right, “lightly” castrated animals were deemed and are deemed stronger and more suitable as working animals than “heavily” castrated reindeer. A lightly castrated animal would still attempt to rub off the velvet from its antlers before the rut and might even participate in the rut if gone wrong. This, however, is not considered desirable as these animals are thought to be more dangerous than fully or lightly castrated animals—less scared of people and more aggressive during the rut (Paine 1994, pp 26–28). More heavily or fully castrated individuals are considered to tire quickly, be lazier, and be less willing to work (e.g., Wustmann 1951; Skjenneberg and Slagsvold 1979, p 281). Since the ban of the gasket method and the widespread introduction of large-scale reindeer pastoralism focused on meat production, the practices surrounding castration strength became obsolete in some areas. In others, however, the tradition is still very much alive. Different techniques are performed with pliers to achieve the desired castration strength, mainly used for tourist and racing reindeer (Van den Berg 2022, unpublished manuscript).

### The effect of castration on bones

*Rangifer tarandus tarandus* reach sexual maturity between 18 and 30 months (Skjenneberg 1984) and skeletal maturity at about 4.5 years of age (Hufthammer 1995; Takken Beijersbergen and Hufthammer 2012). Longitudinal bone growth is linked to epiphyseal fusion (Kennedy et al. 1999), and in general, bones cease longitudinal growth once epiphyseal fusion is complete (e.g., Silver 1963). Bones may reach their maximum length sometime before epiphyseal fusion (Moran and O’Connor 1994; Popkin et al. 2012). Bone growth in the breadth and depth dimensions may also conclude before fusion or advance following fusion (Payne and Bull 1988; Davis 1996, 2000), or, in some rare cases, shrinkages might occur post-fusion (Davis 1996, 2000).

In general, females fuse their elements earlier than males, which affects bone size (Moran and O’Connor 1994; Davis 2000; Popkin et al. 2012). Also reindeer are a sexually dimorphic species, and distinct sex differences in bone size have been marked in earlier studies, with male bones being notably larger than female bones (e.g., Reimers et al. 1983; Weinstock 2000b, 2002; Puputti and Niskanen 2009; Pelletier et al. 2020).

For several studied species, the castrates show a clear pattern of delayed epiphyseal fusion relative to both males and females (Noddle 1974; Hatting 1983; Moran and O’Connor 1994; Davis 2000). Castration is thought to allow the elongation of the bones in several studied species and the long bones in particular (Hobday 1914; Silberberg and Silberberg 1971; Davis 2000). This elongation of the bones would result in an overall increased “slenderness” of the bones of castrates (Hammond 1932; Bradfield 1967; Brannang 1971; Kay and Houseman 1974; Davis 2000). However, the effect of castration on bone size appears to be more complex, as Popkin et al. (2012) found in their study on Shetland sheep through osteometric analysis. Though castrated sheep show a delay in epiphyseal fusion relative to males and females, the effect did not necessarily ensue in an elongation of the long bones. They found that castrated sheep often appeared female in size and shape, and in many cases, bones held characteristics of both males and females, depending on the osteometric measurement.

It is known from anthropological studies that castration is indeed thought to affect skeletal development in reindeer (Silberberg and Silberberg 1971; Skjenneberg and Slagsvold 1979, p 283; Van den Berg 2022, unpublished manuscript). The age of castration has a profound effect on the development of the reindeer, as castration at an early age halts the development of the reindeer (Skjenneberg and Slagsvold 1979, p 283). The effects of castration are more evident in the development of the skeleton when animals are castrated at a younger age than at a more advanced age (e.g., Telldahl et al. 2012). As castration affects the epiphyseal fusion of the bones and, therefore, bone growth, it is unlikely that castration can be detected from the size and form of the bones if it is done after epiphyseal fusion is complete and skeletal maturity is reached.

## Materials and methods

### The reindeer sample

In this study, we measured 298 complete or partial limb bones (humerus, radioulna, metacarpus, femur, tibia, and metatarsus) and pelvises (Table 1) of 97 reindeer individuals from the Fennoscandian domestic reindeer (*Rangifer tarandus tarandus*). Limb bones or limb bone fragments are relatively durable, commonly found in archaeological sites, and easily identifiable at the species level (Puputti and Niskanen 2009). We did not include the same elements from the same individuals; either the left or right elements of one individual were used. All specimens used in this study are of known sex and castration status. For the purpose of our analyses, we divided our sample into three groups based on sex and castration: Group 1 are castrated males (*n* = 30), group 2 are full males (*n* = 27), and group 3 are females (*n* = 40). We visited and measured bones from the reindeer bone collections of the Biodiversity Unit of the University of Oulu, Finland, the University Museum of the University of Bergen, Norway, and the Ájtte Swedish Sámi and Mountain Museum, Sweden. These collections are currently archived at the said institutions. The collections were visited in the period between summer 2019 and autumn 2020.

**Table 1:** We measured 298 complete and partial limb bones and pelvises from the collections of the University of Oulu, the University of Bergen, and the Ájtte Museum

Element	Group			Total
Collection	Castrates	Full males	Females	
Humerus	9	8	14	31
University Museum of Bergen		1	3	4
University of Oulu	9	7	11	27
Radioulna	9	7	16	32
University Museum of Bergen		1	3	4
University of Oulu	9	6	13	28
Metacarpus	25	24	31	80
Ájtte Museum	3	10	14	27
University Museum of Bergen		2	3	5
University of Oulu	22	12	14	48
Femur	8	8	13	29
University Museum of Bergen		1	2	3
University of Oulu	8	7	11	26
Tibia	9	9	15	33
University Museum of Bergen		2	3	5
University of Oulu	9	7	12	28
Metatarsus	25	24	32	81
Ájtte Museum	5	10	12	27
University Museum of Bergen		2	3	5
University of Oulu	20	12	17	49
Pelvis	1	2	9	12
University Museum of Bergen		1	2	3
University of Oulu	1	1	7	9
Total	86	82	130	298

The osteological material in this study comes from different reindeer populations. The sample from the collection of the Biodiversity Unit of the University of Oulu comprises individuals from Enontekiö, Hyrynsalmi, Ii, Inari, Ivalo, Kuhmo, Kuusamo, Oulu, Pudasjärvi, Simo, Suomussalmi, and Yli-Ii, collected between 1963 and 2020. The sample from the collection of the University Museum of the University of Bergen contains individuals from Finnmark, Oppland, Svarthøy, and Svondalen, collected between 1869 and 2006. The sample from the collection of the Ájtte Museum holds individuals from Älvsbyn, Funäsdalen, Hotagen sameby, Jänsmässholmens, Könkämä sameby, Luokta-Mavas sameby, Mittådalen, Rödingsträsk skogslappby, and Vittangi sameby, collected between 1952 and 1955.

Our sample for different bone elements generally ranges from 7 to 22 samples, with most samples in the female group and the least in the castrated and full male groups. Because of its small sample size, the pelvis bone was excluded from most (statistical) analyses.

### Age

Most specimens used in this study were adults whose age at death was recorded in the collections. We only included fused bone elements of the specimens with no recorded age. An exception in our study is the pelvic bone, the latest fusing element in the reindeer skeleton, and of which we only had 12 specimens. Some centers of ossification in the pelvis start to fuse around 45 months of age, but others much later, and to this day, it remains unclear when these parts fuse exactly (Takken Beijersbergen and Hufthammer 2012). We decided to include some of the not fully fused pelvic specimens in our study (7 out of 12 were partially fused), taking note of which parts were fused, fusing, or unfused, and reasoning which measurements would be affected by this. For example, if the iliac crest was not fused, we know the “greatest length” measurement would be highly affected, and this measurement was thus excluded from our analysis. In our study, we only included castrated reindeer that were castrated between 3 and 4 years of age or younger. We treated those as one group because of the restricted sample size, regardless of differences in castration ages.

### Osteometric measurements

**Fig. 3:**
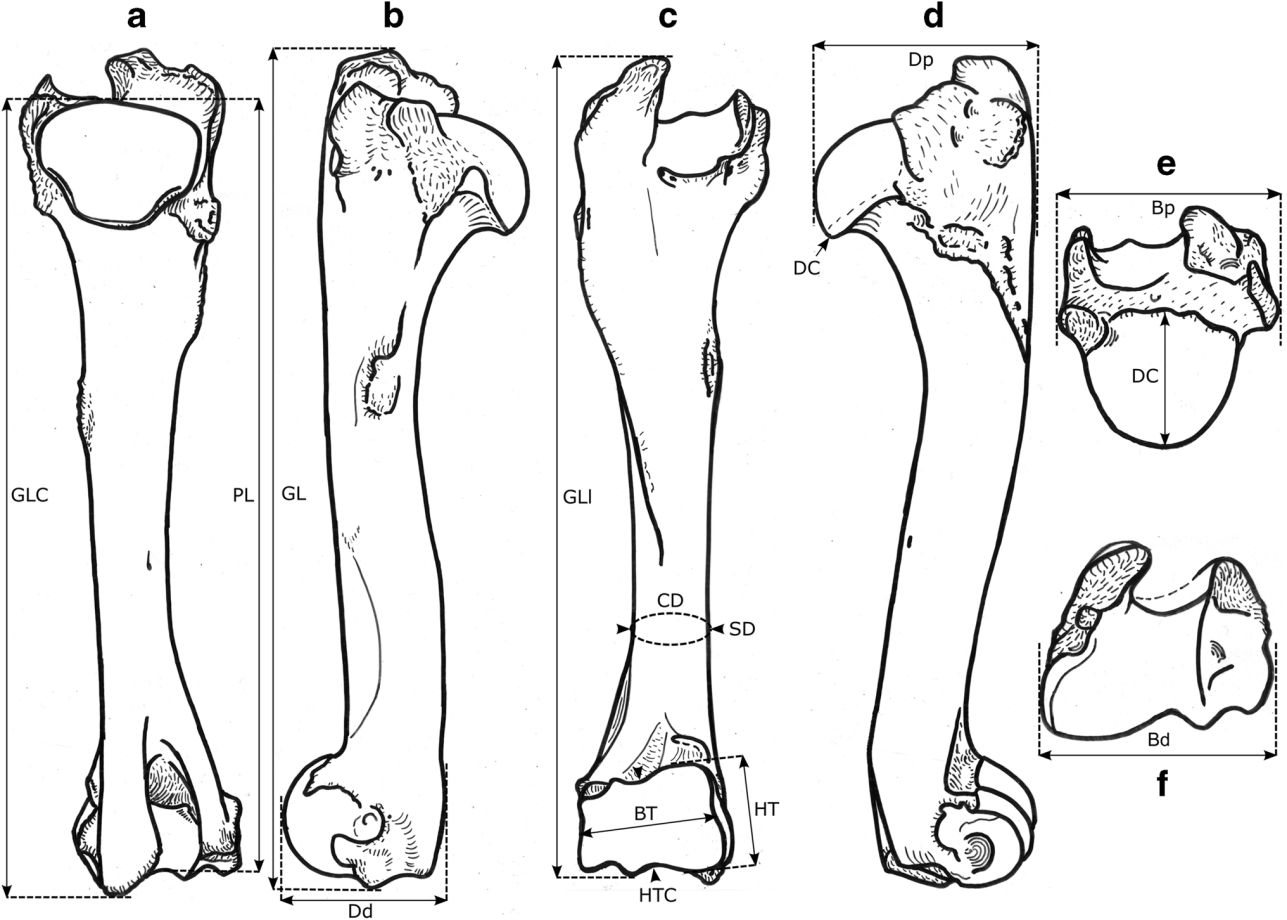
Measurements taken from the humerus (see Table 2 for full definitions). The drawings show a right-side humerus in the a caudal, b medial, c cranial, d lateral, e proximal, and f distal view. (Illustration: Mathilde van den Berg)

**Fig. 4:**
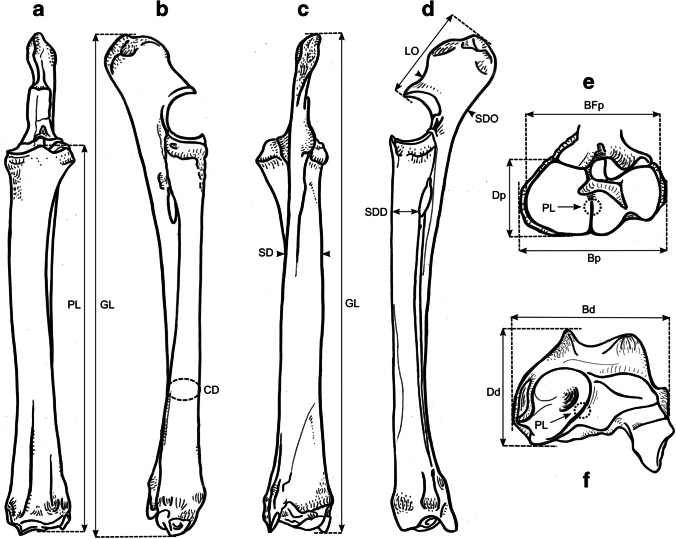
Measurements taken from the radioulna (see Table 2 for full definitions). The drawings show a left-side radioulna in the a dorsal, b medial, c palmar, d lateral, e proximal, and f distal view. (Illustration: Mathilde van den Berg)

**Fig. 5:**
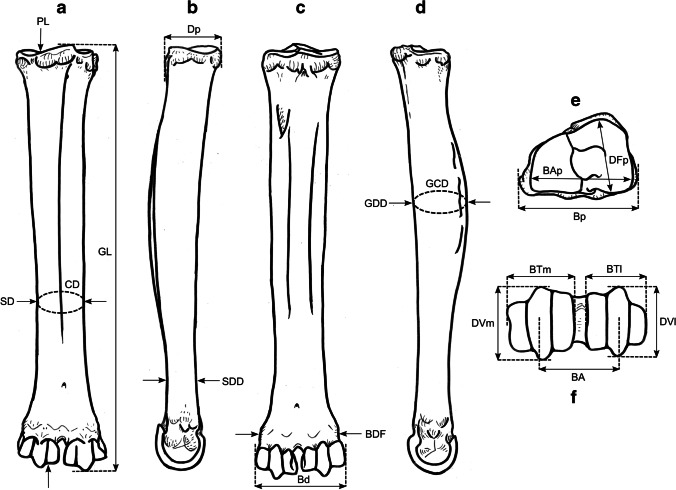
Measurements taken from the metacarpus (see Table 2 for full definitions). The drawings show a left-side metacarpus in the a dorsal, b medial, c palmar, d lateral, e proximal, and f distal view. (Illustration: Mathilde van den Berg)

**Fig. 6:**
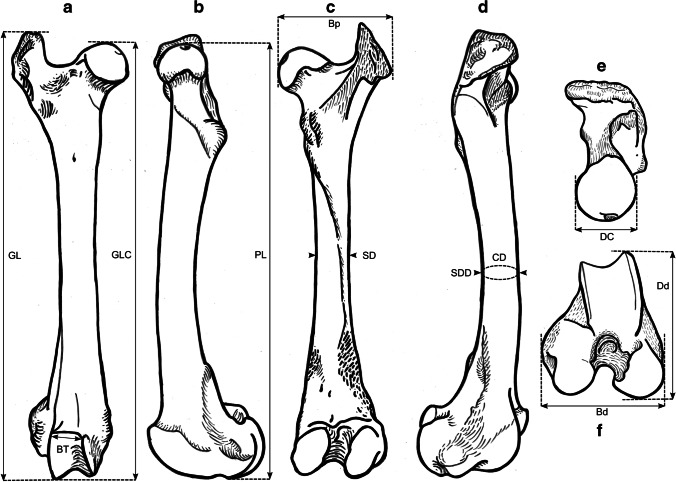
Measurements taken from the femur (see Table 2 for full definitions). The drawings show a right-side femur in a cranial, b medial, c caudal, d lateral, e proximal, and f distal view. (Illustration: Mathilde van den Berg)

**Fig. 7:**
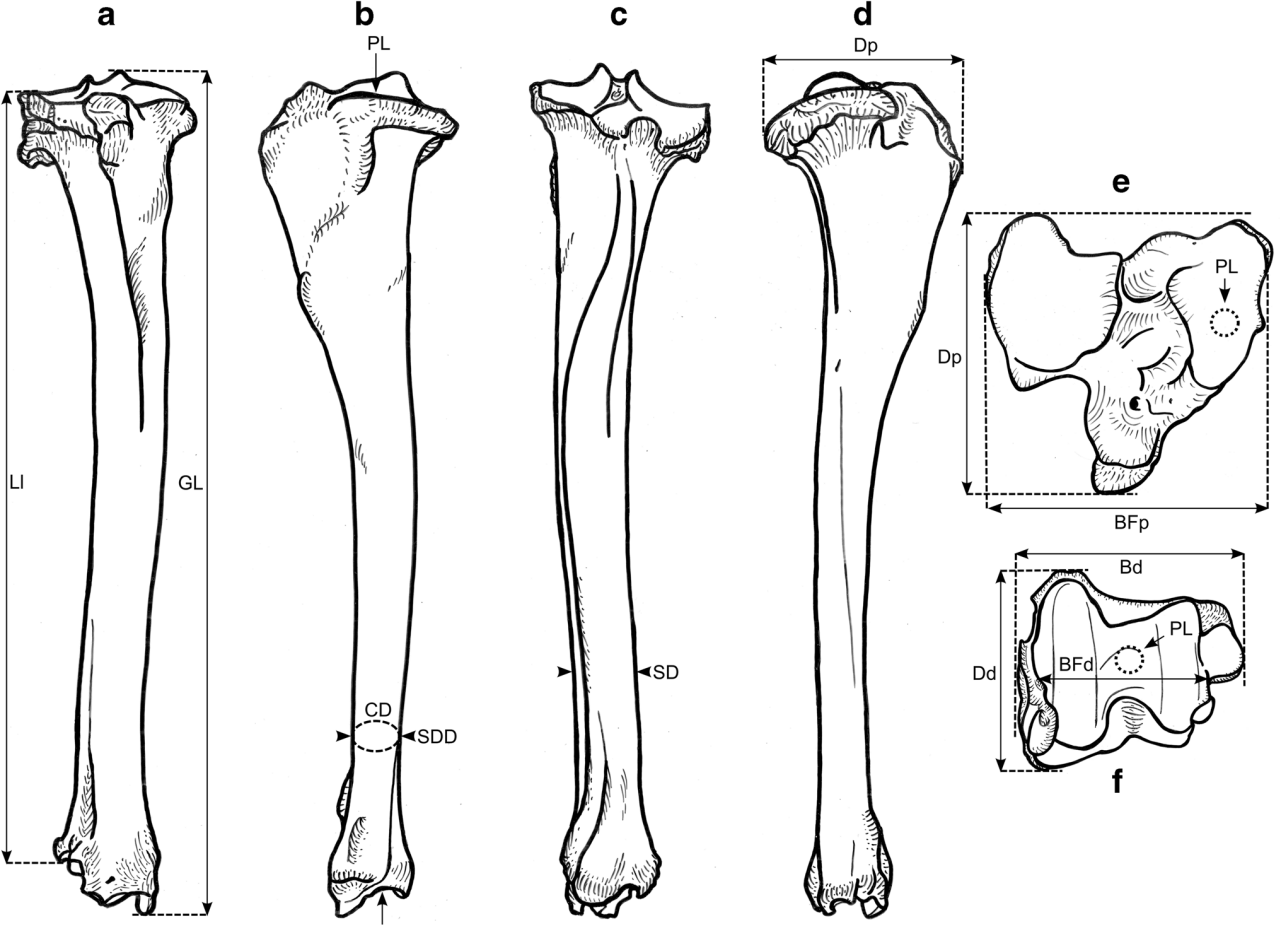
Measurements taken from the tibia (see Table 2 for full definitions). The drawings show a right-side tibia in the a dorsal, b medial, c plantar, d lateral, e proximal, and f distal view. (Illustration: Mathilde van den Berg)

**Fig. 8:**
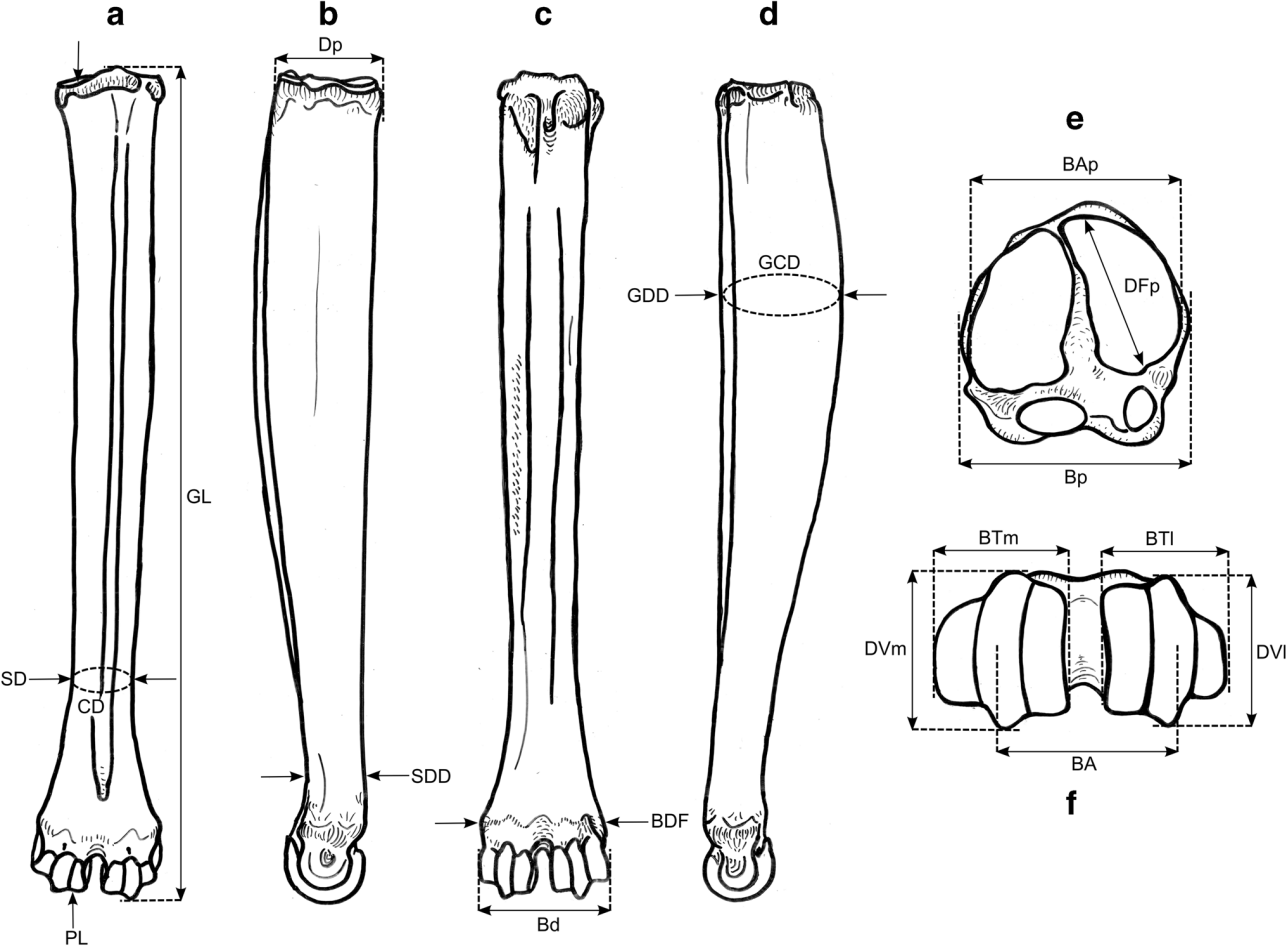
Measurements taken from the metatarsus (see Table 2 for full definitions). The drawings show a left-side metatarsus in the a dorsal, b medial, c plantar, d lateral, e proximal, and f distal view. (Illustration: Mathilde van den Berg)

**Fig. 9:**
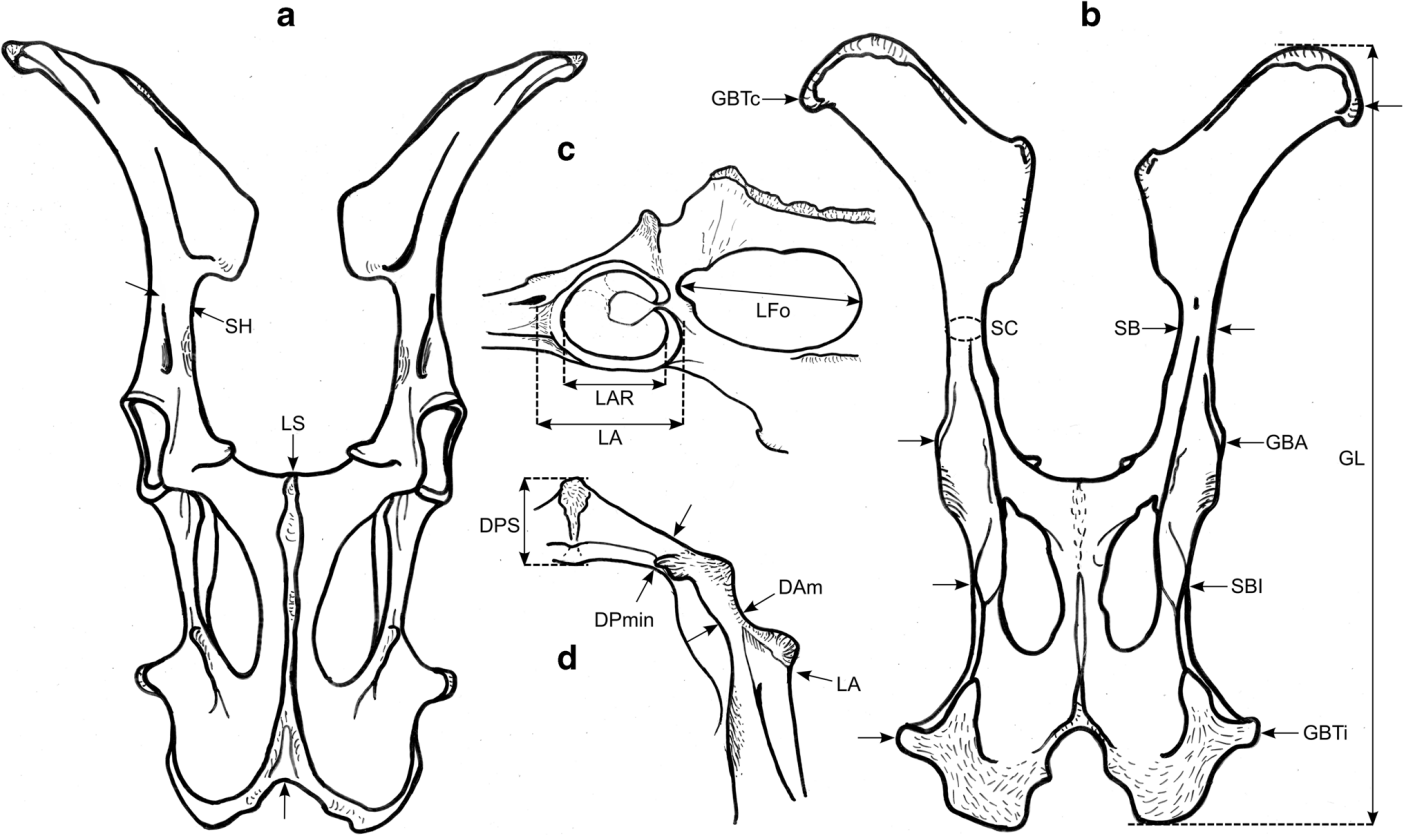
Measurements taken from the pelvis (see Table 2 for full definitions). The drawings show a fused pelvis in the a ventral, b dorsal, c right lateral, and d left cranial view. (Illustration: Mathilde van den Berg)

We took a total of 99 different measurements, most of which have been defined by other authors, and additional measurements were designed for this study. See Table 2 for the complete set of used measurements and their definitions. All measurements were vectorized on reindeer bone drawings (Figs. 3, 4, 5, 6, 7, 8, and 9). The bones were measured with 4 different measuring tools, which were used depending on the type of measurement: a digital caliper (to the nearest tenth of a millimeter), a large-size caliper (to the nearest millimeter), a measuring box (to the nearest millimeter), and tape measure (to the nearest millimeter). We did not obtain a complete set of measurements for some bones (e.g., from the Ájtte Museum collection) because of, e.g., breakage, pathological lesions, or tissue/articular elements attached to the bone.

**Table 2:** Measurement definitions (Mm = measurement), with used measuring device, metric, original reference to the measurement, and original name

Element	Mm	Definition	Metric	Device	Reference	Original name
Humerus	GL	Greatest length. The long axis must lie parallel to the supporting surface. Measured from the caudal major tubercle’s most proximal projection to the distal end’s most distal projection	cm	Measuring box	Von den Driesch 1976	
Humerus	GLC	Greatest length measured from the humeral caput, measured from the proximal flat surface of the caput to the most distal end of the humerus, parallel to the longitudinal axis	cm	Big calipers	Von den Driesch 1976	
Humerus	GLI	Greatest length lateral. Measured from the most proximal projection of the greater tubercle to the most distal projection of the middle ridge of the trochlea, between the lateral and medial condyle in parallel with the longitudinal axis	cm	Big calipers	Von den Driesch 1976	
Humerus	Bp	Greatest breadth of the proximal end. Measured from the most medial projection of the minor tubercle to the most lateral projection of the major tubercle	cm	Measuring box	Von den Driesch 1976	
Humerus	SD	Smallest breadth of the diaphysis. Smallest mediolateral diameter of the diaphysis. Measured perpendicular to the longitudinal axis	mm	Small calipers	Von den Driesch 1976	
Humerus	CD	Smallest circumference of the diaphysis. Measured perpendicular to the longitudinal axis	cm	Tape measure	This study	
Humerus	Bd	Greatest breadth of the distal end. Greatest breadth from the most medial projection of the medial trochlea, beyond the direct articular surface, to the most lateral projection of the lateral trochlea, not including the lateral epicondyle	cm	Measuring box	Von den Driesch 1976	
Humerus	BT	Greatest breadth of the trochlea. Measured from the most lateral point of the lateral trochlea to the most medial point of the medial trochlea, at the point of the most cranial projection, measured not at a right angle to the longitudinal axis of the humerus but to the imagined mediolateral axis of the trochlea. The epicondyles are not included into this measurement	mm	Small calipers	Von den Driesch 1976	
Humerus	HT	Height of the trochlea. The height of the medial condyle (trochlea) is measured not at a right angle to the longitudinal axis of the humerus but to the imagined mediolateral axis of the trochlea	mm	Small calipers	Puputti and Niskanen 2008	HUM TH
Humerus	DC	Anterior–posterior depth of the caput. Measured from the most cranial point of the flat articular surface of the humeral caput to the most caudal point of the caudal-distal rim of the caput	mm	Small calipers	Puputti and Niskanen 2008	HUM HHAP
Humerus	HTC	Height trochlear constriction. Vertical diameter of the central trochlear constriction, or depression between the lateral and medial condyle, as parallel to the longitudinal axis of the humerus	mm	Small calipers	Davis 1996	
Humerus	Dp	Greatest depth of the proximal end. Anterior–posterior depth from the most caudal projection of the humeral caput to the most cranial projection of the most distal bulge of the major tubercle, measured perpendicular to the longitudinal axis of the humerus	cm	Measuring box	Von den Driesch 1976	
Humerus	Dd	Greatest depth of the distal end. Anterior–posterior depth from the most caudal projection of the medial epicondyle to the most cranial point of the medial trochlea. Dd is defined differently by Weinstock 2000a	mm	Small calipers	This study	
Humerus	PL	Physiological length. Measured parallel to the longitudinal axis of the humerus from the most distal projection of the flat humeral articular facet to the central constriction of the trochlea	cm	Big calipers	This study	
Radioulna	GL	Greatest length of the radioulna. Measured from the most proximal projection of the tubercle of the olecranon to the most distal projection of the styloid process of either the radius or ulna (depending on which is most distal). Measured parallel to the longitudinal axis	cm	Measuring box	Von den Driesch 1976	
Radioulna	PL	Physiological length of the radius. The longitudinal axis measured from the middle of the lateral ridge of the medial articular facet of the proximal radius to the most distal ridge between the articular surfaces where the radius articulates with the carpal bones	cm	Curved calipers	Niinimäki et al. 2021	LI
Radioulna	Bp	Breadth of the proximal end of the radius. Measured from the most lateral process to the most medial process of the proximal radius, i.e., including tubercles	mm	Small calipers	Von den Driesch 1976	
Radioulna	BFp	Greatest breadth of the proximal articular facet of the radius. Measured in the same plane as the Bp	mm	Small calipers	Von den Driesch 1978	
Radioulna	CD	Smallest circumference of the diaphysis of the radioulna. Measured perpendicular to the longitudinal axis	cm	Tape measure	Von den Driesch 1979	
Radioulna	Bd	Greatest breadth of the distal end of the radioulna. Measured from the most medial process of the distal radius to the most lateral projection of the tubercle of the ulnar styloid process, or the most lateral tubercle of the distal radius	mm	Small calipers	This study	
Radioulna	SD	Smallest breadth of the radial diaphysis. Smallest mediolateral diameter, excluding the ulna	mm	Small calipers	Von den Driesch 1979	
Radioulna	SDD	Smallest depth of the radial diaphysis. Usually measured dolsar-palmar at the height of the antebrachial interosseous space	mm	Small calipers	Von den Driesch 1979	
Radioulna	Dd	Depth of the distal end. Measured from the most dorsal projections of the radial medial and lateral tubercles to the most palmar projection of the distal radial, above the radial articular facets	mm	Small calipers	Weinstock 2000a	
Radioulna	Dp	Depth of the proximal end. Depth of the medial articular facet	mm	Small calipers	Puputti and Niskanen 2009	RAD PRAP
Radioulna	LO	Length of the olecranon. Measured from the most proximal projection of the tubercle olecranon to the most distal notch of the processus anconeus (or most dorsal notch of the trochlear incisura), measured in parallel to the olecranon’s “own” imaginary axis	mm	Small calipers	Von den Driesch 1976	
Radioulna	SDO	Smallest depth of the olecranon. Measured from the most dented point on the dorsal ridge to the palmar ridge of the olecranon	mm	Small calipers	Von den Driesch 1976	
Metacarpus	GL	Greatest length. Measured in the longitudinal axis from the most proximal projection of the articular surface to the most distal projection of the verticuli of the trochlei	cm	Measuring box	Von den Driesch 1976	
Metacarpus	Bp	Breadth proximal. Maximum medial–lateral diameter of the proximal end, including the tubercles	mm	Small calipers	Von den Driesch 1976	
Metacarpus	Dp	Depth proximal. Maximum dorsal-palmar diameter of the proximal end, including the tubercles	mm	Small calipers	Von den Driesch 1976	
Metacarpus	SD	Smallest breadth of the diaphysis. Smallest mediolateral diameter of the diaphysis	mm	Small calipers	Schild 1962; Von den Driesch 1976	
Metacarpus	CD	Smallest circumference of the diaphysis. Measured perpendicular to the longitudinal axis	cm	Tape measure	Von den Driesch 1976	
Metacarpus	Bd	Breadth distal. The maximum medial–lateral diameter of the distal end is measured from the most lateral projections of the medial and lateral epicondyles. Perpendicular to the longitudinal axis	mm	Small calipers	Von den Driesch 1976	
Metacarpus	BTm	Breadth of the medial trochlea. Greatest medial–lateral diameter of the medial trochlea, perpendicular to the longitudinal axis	mm	Small calipers	Telldahl et al. 2012	BFdm
Metacarpus	BTl	Breadth of the lateral trochlea. Greatest medial–lateral diameter of the lateral trochlea, perpendicular to the longitudinal axis	mm	Small calipers	Telldahl et al. 2012	BFdl
Metacarpus	DVm	Depth of the medial verticulus. Greatest dorsal-palmar diameter of the medial verticulus	mm	Small calipers	Davis 1996; SGWP in Popkin et al. 2012	DVM
Metacarpus	DVl	Depth of the lateral verticulus. Greatest dorsal-palmar diameter of the lateral verticulus	mm	Small calipers	Davis 1996; SGWP in Popkin et al. 2012	DVL
Metacarpus	BAp	Breadth of the proximal articular surface. Greatest medial–lateral diameter of the proximal articular facets, perpendicular to the longitudinal axis	mm	Small calipers	SGWP in Popkin et al. 2012	BFP
Metacarpus	BDF	Breadth of the diaphysis along the distal line of fusion. Greatest medial–lateral diameter of the fusion site of the distal end, including tubercles	mm	Small calipers	Popkin et al. 2012	BdFus
Metacarpus	BA	Breadth between the articular crests. Measured between the most distal points of the crests	mm	Small calipers	Telldahl et al. 2012	Bcr
Metacarpus	GCD	Greatest circumference of the diaphysis. Measured perpendicular to the longitudinal axis	cm	Tape measure	This study	
Metacarpus	GDD	Greatest depth of the diaphysis. Measured perpendicular to the longitudinal axis	mm	Small calipers	This study	
Metacarpus	SDD	Smallest depth of the diaphysis. Smallest dorsal-palmar diameter of the diaphysis	mm	Small calipers	This study	
Metacarpus	PL	Physiological length. Measured from the most distal point of the proximal medial articular surface to the most distal projection of the lateral epicondyle of the medial trochlea	cm	Curved calipers	This study	
Metacarpus	DFp	Depth of the proximal articular facet. Measured from the medial facet, not perpendicular to the longitudinal axis, nor strictly dorsal-palmar, but as shown in Fig. 5e	mm	Small calipers	This study	
Femur	GL	Greatest length. Measured from the most proximal projection of the major trochanter to the most distal projection of the trochlear ridge(s) and/or condyle(s) (depending on which point is most distal, which might vary between individuals), parallel to the longitudinal axis of the femur. The bone must not lie flat on the measuring board but with its proximal end slightly raised	cm	Measuring box	Von den Driesch 1976	
Femur	GLC	Greatest length measured from the most proximal point of femoral caput to the most distal projection of the trochlear ridge(s) and/or condyle(s) (depending on which point is most distal, which might vary between individuals), parallel to the longitudinal axis of the femur	cm	Measuring box	Von den Driesch 1976	
Femur	Bp	Breadth of the proximal end. Measured from the most lateral projection of the major trochanter to the most medial point of the femoral caput. Measured perpendicular to the longitudinal axis	mm	Small calipers	Von den Driesch 1976	
Femur	DC	Depth of the femoral caput. Greatest cranial-caudal diameter of the femoral caput in dorsal-plantar. Perpendicular to the longitudinal axis	mm	Small calipers	Von den Driesch 1976	
Femur	SD	Smallest breadth of the diaphysis. Smallest mediolateral diameter of the diaphysis. Perpendicular to the longitudinal axis	mm	Small calipers	Von den Driesch 1976	
Femur	SDD	Smallest depth of the diaphysis. Smallest cranial-caudal diameter of the diaphysis. Measured perpendicular to the longitudinal axis	mm	Small calipers	This study	
Femur	CD	Smallest circumference of the diaphysis. Measured perpendicular to the longitudinal axis	cm	Tape measure	Von den Driesch 1976	
Femur	Bd	Breadth of the distal end. Measured from the most lateral projection of the lateral epicondyle to the most medial projection of the medial condyle or epicondyle, depending on which is broader in the individual. Perpendicularly to the longitudinal axis of the femur	mm	Small calipers	Von den Driesch 1976	
Femur	Dd	Depth of the distal end. Greatest cranial-caudal diameter measured from the most cranial projection of the medial trochlear tubercle (ridge) to the most caudal projection of the medial condyle, perpendicular to the longitudinal axis	cm	Measuring box	Weinstock 1997	
Femur	BT	Breadth of the trochlea. Measured horizontally between the trochlea’s medial and lateral ridges, at the trochlea’s most cranial end. BT is Defined differently by Weinstock 2000a	mm	Small calipers	This study	
Femur	PL	Physiological length. Measured between the most proximal point of the femoral caput to the most distal articular surface of the medial condyle, parallel to the longitudinal axis	cm	Big calipers	Niinimäki et al. 2021	LI
Tibia	GL	Greatest length. Measured between the most proximal point(s) of the condyle(s) and the most distal projection of the medial malleolus, parallel to the longitudinal axis	cm	Measuring box	Von den Driesch 1976	
Tibia	Ll	Length lateral. Measured between the most lateral proximal point of the lateral tibular condyle and the most distal lateral projection of the medial malleolar process, parallel to the longitudinal axis	cm	Big calipers	Von den Driesch 1976	
Tibia	SD	Smallest breadth of the diaphysis. Smallest mediolateral diameter of the diaphysis. Perpendicular to the longitudinal axis	mm	Small calipers	Von den Driesch 1976	
Tibia	SDD	Smallest depth of the diaphysis. Smallest dorsal-plantar diameter of the diaphysis. Perpendicular the longitudinal axis	mm	Small calipers	This study	
Tibia	CD	Smallest circumference of the diaphysis. Measured perpendicular to the longitudinal axis	cm	Tape measure	Von den Driesch 1976	
Tibia	Bd	Breadth distal. Measured between the most medial projection of the medial malleolus and most lateral projection of the medial malleolus, perpendicular to the dorsal-plantar axis of the direction of the distal articular grooves	mm	Small calipers	Von den Driesch 1976	
Tibia	Dd	Depth distal. Measured between the most dorsal projection of the distal tibia and the most plantar projection of the distal tibia, parallel to the dorsal-plantar axis of the direction of the distal articular grooves	mm	Small calipers	Von den Driesch 1976	
Tibia	Dp	Depth proximal. Measured between the proximal-most dorsal point of the tibial tuberosity and the most plantar projections of the medial and/or lateral tibial condyle(s) (depending on the individual which projects most plantar), perpendicular to the longitudinal axis of the tibia	cm	Measuring box	Puputti and Niskanen 2009	TIB PTAP
Tibia	PL	Physiological length. Greatest length between the central intercondylar eminence of the proximal end to the mid-ridge of the distal articular facet. Measured parallel to the longitudinal axis	cm	Curved calipers	Niinimäki et al. 2021	LI
Tibia	BFp	Breadth of the proximal articular facet. Measured between the most medial point of the medial condylar surface to the most lateral point of the lateral condylar surface. Measured perpendicular to the longitudinal axis of the tibia	mm	Small calipers	Niinimäki et al. 2021	
Tibia	BFd	Breadth of the distal articular facet. The breadth of the articular facet, which articulates with the talus. The malleolar articular facet of the most lateral face is not included in the measurement. Measured perpendicular to the dorsal-plantar axis of the direction of the distal articular grooves	mm	Small calipers	This study	
Metatarsus	GL	Greatest length. Measured in the longitudinal axis from the most proximal projection of the articular surface to the most distal projection of the verticuli of the trochlei	cm	Measuring box	Von den Driesch 1976	
Metatarsus	Bp	Breadth proximal. Maximum medial–lateral diameter of the proximal end, including the tubercles	mm	Small calipers	Von den Driesch 1976	
Metatarsus	Dp	Depth proximal. Maximum dorsal-plantar diameter of the proximal end, including the tubercles	mm	Small calipers	Von den Driesch 1976	
Metatarsus	SD	Smallest breadth of the diaphysis. Smallest mediolateral diameter of the diaphysis. Measured perpendicular to the longitudinal axis	mm	Small calipers	Schild 1962; Von den Driesch 1976	
Metatarsus	CD	Smallest circumference of the diaphysis. Measured perpendicular to the longitudinal axis	cm	Tape measure	Von den Driesch 1976	
Metatarsus	Bd	Breadth distal. Maximum medial–lateral diameter of the distal end measured from the most lateral projections of the medial and lateral epicondyles. Perpendicular to the longitudinal axis	mm	Small calipers	Von den Driesch 1976	
Metatarsus	BTm	Breadth of the medial trochlea. Greatest medial–lateral diameter of the medial trochlea, perpendicular to the longitudinal axis	mm	Small calipers	Telldahl et al. 2012	BFdm
Metatarsus	BTl	Breadth of the lateral trochlea. Greatest medial–lateral diameter of the lateral trochlea, perpendicular to the longitudinal axis	mm	Small calipers	Telldahl et al. 2013	BFdl
Metatarsus	DVm	Depth of the medial verticulus. Greatest dorsal-plantar diameter of the medial verticulus. Measured perpendicular to the longitudinal axis	mm	Small calipers	Davis 1996; SGWP in Popkin et al. 2012	DVM
Metatarsus	DVl	Depth of the lateral verticulus. Greatest dorsal-plantar diameter of the lateral verticulus. Measured perpendicular to the longitudinal axis	mm	Small calipers	Davis 1996; SGWP in Popkin et al. 2012	DVL
Metatarsus	BAp	Breadth of the proximal articular surface. Greatest medial–lateral diameter of the proximal articular facet, perpendicular to the longitudinal axis	mm	Small calipers	SGWP in Popkin et al. 2012	BFP
Metatarsus	BDF	Breadth of the diaphysis along the distal line of fusion. Greatest medial–lateral diameter of the fusion site of the distal end, including tubercles. Measured perpendicular to the longitudinal axis	mm	Small calipers	Popkin et al. 2012	BdFus
Metatarsus	BA	Breadth between the articular crests. Measured between the most distal points of the crests	mm	Small calipers	Telldahl et al. 2012	Bcr
Metatarsus	GCD	Greatest circumference of the diaphysis. Measured perpendicular to the longitudinal axis	cm	Tape measure	This study	
Metatarsus	GDD	Greatest depth of the diaphysis. Measured perpendicular to the longitudinal axis	mm	Small calipers	This study	
Metatarsus	SDD	Smallest depth of the diaphysis. Smallest dorsal-plantar diameter of the diaphysis	mm	Small calipers	This study	
Metatarsus	PL	Physiological length. Measured from the most distal point of the proximal medial articular surface to the most distal projection of the lateral epicondyle of the medial trochlea	cm	Curved calipers	This study	
Metatarsus	DFp	Depth of the proximal articular facet. Measured from the medial facet, not perpendicular to the longitudinal axis but as shown in Fig. 8e	mm	Small calipers	This study	
Pelvis	GL	Greatest length of one half. Measured from the most cranial projection of the iliac crest to the most caudal projection of the tuber ischiadicum. Important that the epiphyseal parts of the tuber coxae and the tuber ischiadicum have fused	cm	Measuring box	Von den Driesch 1976	
Pelvis	LA	Length of the acetabulum, including the lip. Across the facies lunata, measured in the directions of the ischium and os ilium, including the lip, and in the direction of the os ilium, the measurement is extended to the dent of the lateral musculus rectus femoris attachment site	mm	Small calipers	Von den Driesch 1976	
Pelvis	LAR	Length of the acetabulum on the rim. Across the facies lunata, measured in the directions of the ischium and os ilium, measured on the inside of the rim of the acetabular articular surface with the femoral caput	mm	Small calipers	Von den Driesch 1976	
Pelvis	LS	Length of the symphysis. Only when the two pelvic halves have not fused	mm	Small calipers	Von den Driesch 1976	
Pelvis	SH	Smallest height of the shaft ilium. The maximum dorsal–ventral diameter on the most constricted site of the shaft ilium	mm	Small calipers	Von den Driesch 1976	
Pelvis	SB	Smallest breadth of the shaft ilium. The minimum medial–lateral diameter on the most constricted site of the shaft ilium	mm	Small calipers	Von den Driesch 1976	
Pelvis	SC	Smallest circumference of the shaft ilium	cm	Tape measure	Von den Driesch, 1976	
Pelvis	LFo	Inner length of the foramen obturatum. Measured across from the most cranial point close to the acetabulum to the most caudal point towards the ischium	mm	Small calipers	Von den Driesch, 1976	
Pelvis	GBTc	Greatest breadth across the tubera coxaerum. Only when the two pelvic halves have fused	cm	Measuring box	Von den Driesch, 1976	
Pelvis	GBA	Greatest breadth across the acetabula. Measured from the most lateral projections. Only when the two pelvic halves have fused	cm	Measuring box	Von den Driesch, 1976	
Pelvis	GBTi	Greatest breadth across the tubera ischiadica. Only when the two pelvic halves have fused	cm	Measuring box	Von den Driesch, 1976	
Pelvis	SBl	Smallest breadth across the bodies of the ischia. Only when the two pelvic halves have fused	mm	Small calipers	Von den Driesch, 1976	
Pelvis	DPmin	Minimum diameter of the pubis shaft measured in dorsal–ventral direction	mm	Small calipers	Davis, 1996	SHPu
Pelvis	DAm	Depth of the medial rim of the acetabulum. Measured as the ventral-medial border of the acetabulum. It is essential to consider that the rim is pronounced in some individuals while vague in others; do not measure towards the “bulge” resulting from the negative projection of the acetabulum	mm	Small calipers	Davis, 1996	MRDA
Pelvis	DPS	Greatest depth of the pubic symphysis. Along the dorsal–ventral axis	mm	Small calipers	This study

**Fig. 10:**
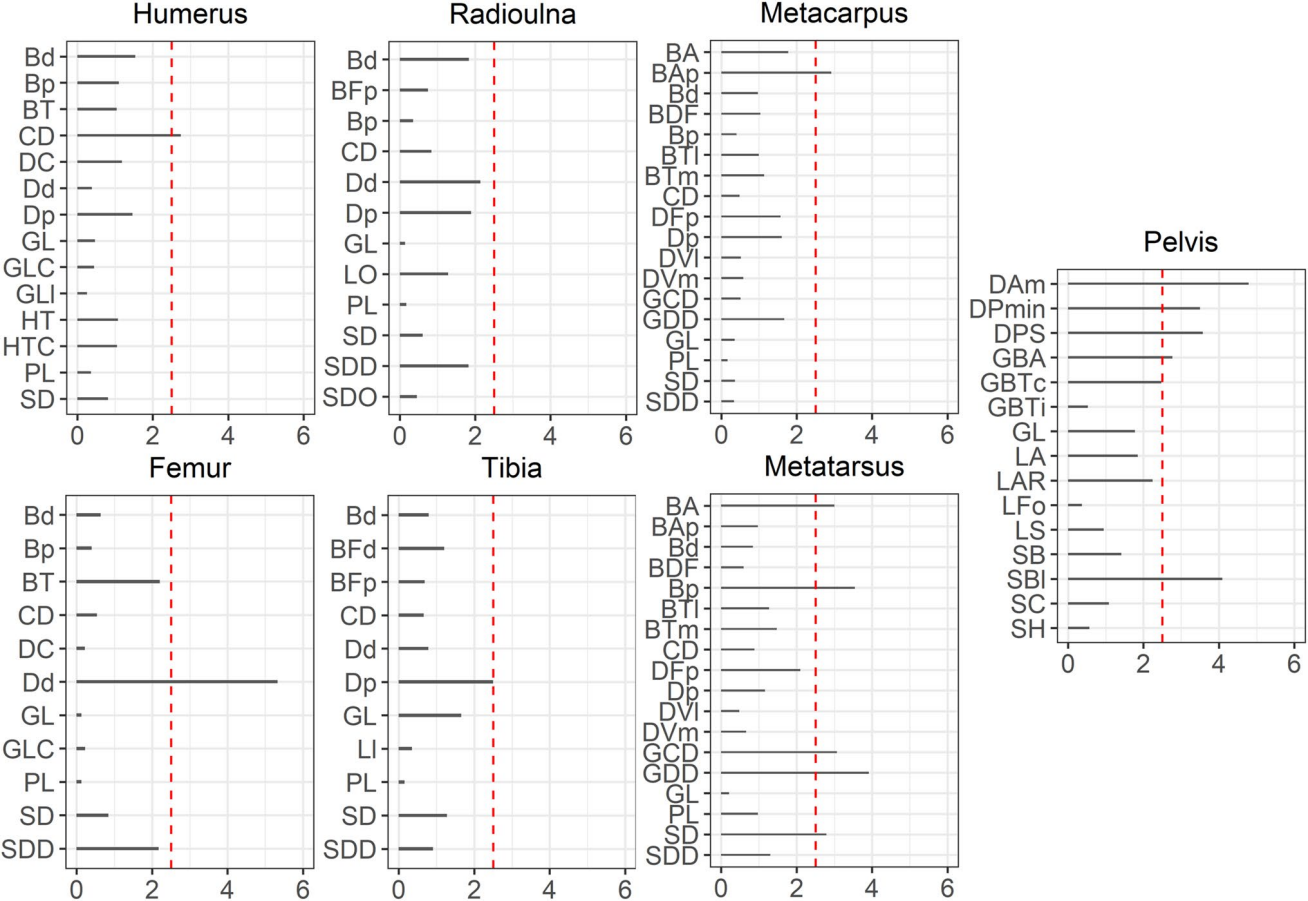
The intra-observer measurement error is presented as the average % difference for all the measurements taken from the long bones and the pelvis

Every biometric measure is associated with measurement error. Measurement error can seriously bias (statistical) analysis when the magnitude of true between-individual variance is small in, e.g., biometric variation within species or populations (Arnqvist and Mårtenson 1998). In our study, intra-observer measurement error was tested on 20 complete bones for each different bone element for all measurements to ensure the accuracy of the recording protocol (Fig. 10). The 20 bones of each element were measured on 4 separate days (day 1, day 2, day 4, and day 7). Following the definition by Harrell and Slaughter (2020, 16–2), intra-observer error was calculated as the mean absolute difference between the measurements from the same observer.

### Analysis and statistical methods

#### Simple variable combinations and Mennerich’s indices

We plotted different measurement variables against each other to achieve metric separation of the groups. For instance, the breadth trochlea (BT) against the breadth proximal (Bp) of the humerus. We discuss only a portion of these results as the numerous different variables for each element and their combinations produced a vast bulk of plots.

By reason of the observation in other ungulate species that castrated males exhibit longer and more slender long bones (e.g., Davis 2000; Shahin et al. 1992), we employed Mennerich’s indices (1968). We plotted Mennerich indices 1 and 3 by calculating the smallest diaphysis breadth (SD) × 100/greatest length (GL) and distal breadth (Bd)/greatest length (GL) × 100, respectively. These indices have shown good results when used, for instance, on cattle metapodials (Telldahl et al. 2012). The results will present the slenderness of the elements as a relation between the greatest length (GL), the diaphysis breadth (SD), and the distal breadth (Bd).

#### Software packages used for statistical analyses

Data manipulation, visualizations, and statistical modeling were conducted in the R programming environment (R Core Team, 2021). Data manipulation was done using package dplyr (Wickham et al. 2021) and visualizations with package ggplot2 (Wickham 2016). Packages caret (Kuhn 2021) and penalizedLDA (Witten 2015) were used for fitting and training penalized linear discriminant analysis models.

#### Exploratory analysis

We used average, coefficient of variation (CV), and percent (%) difference between groups to explore the statistical disposition of our dataset and inspect the characteristics between groups per measurement variable.

#### Penalized linear discriminant analysis

We used penalized linear discriminant analysis (pLDA) for training and fitting the classification models. Compared to the standard LDA, pLDA adds regularization to the model coefficients (Hastie et al. 2009, pp 446–449). Given the small sample size of our dataset and the multicollinearity of the variables, we chose to use pLDA instead of regular LDA to avoid overfitting of the models. The pLDA performs well, especially in settings where the variables are highly correlated, and the goal is to obtain a model with a sparse subset of features (Witten and Tibshirani 2011; Hastie et al. 1995). In addition, a small subset of features leads to higher interpretability, which is highly important in our research setting.

Since our data set is relatively small, we used resampling methods instead of test/train split. We used repeated cross-validation for estimating model performance. The fitted models were evaluated by calculating model accuracy, balanced accuracy,^1^ and F1 Score.^2^ We fitted three types of penalized LDA models: (1) between the three groups, (2) between only castrates versus full males, and (3) between castrates versus full males + females. Predictor importance for each model (for every element) was estimated using ROC curve analyses.

#### Variable correlations, multicollinearity, and variable reduction

We used our results on the correlations between measurements (the “Measurement correlation and linear discriminant analysis” section) to solve the multicollinearity issue of our penalized LDA models. Multicollinearity among variables leads to problems in the prediction and classification ability of the model and produces reliability issues due to overfitting. These problems might be omitted by selecting variables that are less or least correlated with each other (Næs and Mevik 2001). In addition to using correlation analysis for subsetting the features, we used penalized linear discriminant (pLDA) analysis for handling multicollinearity.

With our results on the correlation between measurements (Fig. SI1 to SI6 in the Supplementary Information), we selected a subset of variables for each element for our pLDA (Table SI1 in the Supplementary Information). For complete bones, this serves the purpose of solving the collinearity issue and defining a small group of measurements that are quick and easy to obtain that describe the bone osteometric features as well as possible. Hence, data collection from archaeological reindeer bone assemblages in future applications of our method is quicker and easier. In complete bone analysis, in this way, we could, for example, reduce the number of measurements for the humerus element from 14 to 8 separate measurements. The subsets of collected variables contained at least one variable from each axis (length, depth, breadth, and circumference) and at least one variable from the distal and proximal parts of the bone.

We performed a second form of a reduction on our variable set for each bone element. This reduction involved the selection and analysis of variables of distal and proximal bone parts separately (Table SI1 in the Supplementary Information). Developing a method that works separately for proximal and distal ends is essential because archaeological bone assemblages frequently consist of broken bone fragments (e.g., Puputti and Niskanen 2009). In these selections, we did not omit highly correlated variables for two reasons: (1) the subset of proximal and distal measurements was already so small that we did not want to lose more information, despite high correlation, and (2) multicollinearity presents less trouble in pLDA when the amount of variables is reduced, especially concerning the number of samples in each group.

With our reduced sets of variables of complete bones and separate proximal and distal bone parts, we can advance and attempt to divide the main population into three different groups based on LDA analysis of their biometric traits. We highlighted the two most important variables for the model by every element and elemental part.

## Biometric and statistical results and discussion

### Measurement error

We found that most of the measurements fell within the acceptable limit of our chosen 2.5% difference. Some measurements fell between the 2.5 and 3.75% difference mark, with only one measurement seriously surpassing that threshold: the measurement of the distal depth of the femur (Dd) has a % difference of 5.2. The error must be considered when interpreting the size differences between castrated, full male, and female reindeer bones. The pelvis has several measurements that fall over our 2.5% difference threshold, with the depth of the medial rim of the acetabulum (Dam) and the smallest breadth across the bodies of the ischia (SBI) demonstrating the largest errors. The pelvis measurements have also been found to be the most challenging to record in other osteometric studies (Popkin et al. 2012).

### Differences between the groups

The main questions we are dealing with are which osteometric characteristics differ between castrated males and the other two groups and which variables might be useful for distinguishing castrated, full male, and female reindeer. Descriptive statistics are presented in Table 3.

**Table 3:** The number of complete or partial bones (*N*), coefficient of variation (*CV*), mean, and the % difference per variable between groups. The cells that are left blank (#N/A) for the pelvis indicate insufficient measurements for statistical analysis (*C* = castrate; *M* = full male; *F* = female)

Element	Variable	Castrate			Full male			Female			% Difference		
		*N*	Mean	*CV*	*N*	Mean	*CV*	*N*	Mean	*CV*	*C-M*	*C-F*	*M-F*
Humerus	Bd	9	5.09	0.03	8	5.1	0.04	14	4.5	0.03	− 0.2	13.1	13.3
Humerus	Bp	9	6.10	0.03	8	6.3	0.06	14	5.3	0.03	− 3.6	14.7	18.3
Humerus	BT	9	43.85	0.03	8	44.4	0.04	14	39.3	0.03	− 1.2	11.0	12.2
Humerus	CD	9	8.82	0.07	8	9.0	0.1	14	7.0	0.03	− 2.3	23.0	25.3
Humerus	DC	9	44.16	0.04	8	45.8	0.04	14	39.5	0.04	− 3.7	11.2	14.9
Humerus	Dd	9	49.66	0.04	8	50.6	0.02	14	44.9	0.03	− 1.9	10.2	12.0
Humerus	Dp	9	6.46	0.04	8	6.6	0.05	14	5.6	0.06	− 2.2	14.4	16.6
Humerus	GL	9	25.27	0.03	8	25.7	0.04	14	22.5	0.03	− 1.6	11.6	13.2
Humerus	GLC	9	23.44	0.03	8	23.9	0.04	14	21.0	0.03	− 1.9	11.1	13.0
Humerus	GLl	9	25.07	0.03	8	25.4	0.03	14	22.3	0.03	− 1.2	11.6	12.8
Humerus	HT	9	36.11	0.03	8	37.1	0.04	14	32.5	0.04	− 2.8	10.7	13.4
Humerus	HTC	9	26.93	0.03	8	27.5	0.03	14	24.0	0.04	− 2.2	11.4	13.6
Humerus	PL	9	23.01	0.03	8	23.3	0.03	14	20.6	0.04	− 1.4	11.2	12.6
Humerus	SD	9	24.86	0.07	8	25.5	0.1	14	20.0	0.04	− 2.6	21.4	24.0
Radioulna	BFp	9	44.90	0.04	7	46.3	0.03	16	39.9	0.03	− 3.0	11.7	14.7
Radioulna	Bp	9	49.33	0.06	7	50.4	0.03	16	42.4	0.04	− 2.1	15.1	17.2
Radioulna	CD	9	8.78	0.08	7	9.2	0.07	16	6.7	0.05	− 4.7	26.2	30.8
Radioulna	Dd	9	32.84	0.05	7	34.4	0.05	16	27.8	0.06	− 4.6	16.5	21.0
Radioulna	Dp	9	27.95	0.04	7	28.4	0.02	16	24.3	0.02	− 1.4	13.9	15.4
Radioulna	PL	9	26.10	0.03	7	26.9	0.02	16	23.2	0.02	− 3.0	11.6	14.6
Radioulna	SD	9	29.05	0.09	7	30.8	0.08	16	21.6	0.05	− 5.9	29.5	35.2
Radioulna	SDD	9	18.01	0.06	7	19.4	0.06	16	17.9	0.06	− 7.5	0.9	8.3
Radioulna	Bd	9	47.61	0.06	7	48.1	0.02	16	39.4	0.03	− 1.1	18.9	20.0
Radioulna	GL	9	33.72	0.03	7	34.8	0.02	16	29.8	0.02	− 3.2	12.4	15.6
Radioulna	LO	9	63.17	0.03	7	66.5	0.03	16	53.0	0.03	− 5.1	17.5	22.5
Radioulna	SDO	9	40.79	0.05	7	42.8	0.05	16	34.8	0.04	− 4.8	15.8	20.5
Metacarpus	BA	22	24.44	0.03	14	25.0	0.03	17	22.5	0.04	− 2.4	8.2	10.7
Metacarpus	BAp	22	33.09	0.04	14	34.0	0.06	17	29.8	0.02	− 2.7	10.6	13.3
Metacarpus	Bd	22	44.48	0.03	14	45.2	0.03	17	40.1	0.03	− 1.7	10.5	12.2
Metacarpus	BDF	22	41.50	0.04	14	42.0	0.03	17	35.8	0.04	− 1.2	14.7	15.9
Metacarpus	Bp	22	36.18	0.04	14	36.8	0.04	17	32.1	0.02	− 1.8	12.0	13.8
Metacarpus	BTl	22	20.21	0.04	14	20.5	0.04	17	18.3	0.03	− 1.6	9.9	11.5
Metacarpus	BTm	22	20.93	0.03	14	21.2	0.03	17	18.7	0.03	− 1.3	11.0	12.3
Metacarpus	CD	22	7.31	0.05	14	7.2	0.05	17	5.9	0.06	1.0	22.2	21.2
Metacarpus	DFp	22	23.57	0.04	14	24.0	0.04	17	21.3	0.05	− 1.9	10.2	12.1
Metacarpus	Dp	22	26.40	0.05	14	26.7	0.04	17	23.5	0.04	− 1.2	11.7	12.9
Metacarpus	DVl	22	22.56	0.04	14	22.5	0.03	17	20.6	0.03	0.4	9.3	8.9
Metacarpus	DVm	22	23.03	0.04	14	23.0	0.03	17	20.8	0.03	0.2	10.1	9.9
Metacarpus	GCD	22	8.52	0.08	14	8.5	0.07	17	6.8	0.05	− 0.1	22.7	22.8
Metacarpus	GDD	22	24.87	0.09	14	24.8	0.05	17	19.8	0.05	0.1	22.9	22.8
Metacarpus	GL	22	19.78	0.03	14	19.9	0.03	17	18.2	0.03	− 0.7	8.4	9.1
Metacarpus	PL	22	19.47	0.03	14	19.6	0.03	17	17.9	0.04	− 0.7	8.5	9.2
Metacarpus	SD	22	24.63	0.08	14	24.0	0.08	17	18.8	0.07	2.7	26.9	24.3
Metacarpus	SDD	22	16.53	0.06	14	16.4	0.04	17	13.5	0.05	0.9	19.9	19.0
Femur	Bd	8	62.27	0.05	8	63.0	0.04	13	55.4	0.03	− 1.2	11.8	13.0
Femur	Bp	8	75.14	0.03	8	76.2	0.05	13	64.3	0.03	− 1.4	15.5	16.9
Femur	BT	8	24.16	0.03	8	24.7	0.1	13	21.1	0.04	− 2.2	13.5	15.7
Femur	CD	8	8.36	0.04	8	8.7	0.07	13	6.9	0.03	− 4.0	19.5	23.4
Femur	DC	8	30.43	0.04	8	30.8	0.03	13	27.3	0.02	− 1.3	10.8	12.1
Femur	Dd	8	7.84	0.03	8	7.9	0.06	13	7.0	0.03	− 1.3	10.6	11.9
Femur	GL	8	29.51	0.03	8	29.8	0.03	13	26.3	0.03	− 1.0	11.4	12.4
Femur	GLC	8	28.65	0.03	8	29.1	0.03	13	25.4	0.03	− 1.4	12.1	13.5
Femur	PL	8	28.56	0.03	8	29.0	0.03	13	25.4	0.03	− 1.4	11.8	13.2
Femur	SD	8	24.71	0.06	8	25.6	0.1	13	20.5	0.03	− 3.4	18.7	22.1
Femur	SDD	8	26.81	0.05	8	27.3	0.1	13	21.8	0.04	− 1.8	20.6	22.4
Tibia	Bd	9	43.12	0.04	9	43.7	0.03	15	38.4	0.02	− 1.5	11.5	13.0
Tibia	BFd	9	31.46	0.03	9	31.9	0.03	15	28.2	0.03	− 1.3	11.0	12.4
Tibia	BFp	9	64.43	0.04	9	65.0	0.04	15	56.7	0.05	− 0.9	12.8	13.6
Tibia	CD	9	7.74	0.06	9	7.9	0.08	15	6.4	0.04	− 1.8	19.6	21.5
Tibia	Dd	9	34.44	0.06	9	34.8	0.03	15	31.2	0.04	− 0.9	10.0	10.9
Tibia	Dp	9	6.94	0.05	9	7.1	0.04	15	6.1	0.04	− 2.2	12.7	14.9
Tibia	GL	9	32.96	0.03	9	33.5	0.03	15	29.5	0.03	− 1.6	11.2	12.8
Tibia	Ll	9	30.89	0.03	9	31.3	0.03	15	27.6	0.03	− 1.5	11.1	12.6
Tibia	PL	9	31.63	0.03	9	32.0	0.03	15	28.2	0.03	− 1.3	11.5	12.7
Tibia	SD	9	27.12	0.06	9	27.6	0.09	15	21.6	0.05	− 1.8	22.7	24.5
Tibia	SDD	9	19.99	0.05	9	20.4	0.06	15	17.3	0.05	− 2.0	14.3	16.4
Metatarsus	BA	19	24.28	0.03	14	24.3	0.05	16	22.4	0.06	− 0.2	8.3	8.4
Metatarsus	BAp	19	29.75	0.05	14	29.9	0.03	16	27.2	0.03	− 0.6	8.9	9,5
Metatarsus	Bd	19	43.49	0.03	14	43.9	0.04	16	39.6	0.04	− 0.9	9.3	10.3
Metatarsus	BDF	19	41.86	0.04	14	41.8	0.04	16	36.7	0.04	0.1	13.2	13.1
Metatarsus	Bp	19	32.75	0.05	14	32.7	0.04	16	29.2	0.03	0.1	11.5	11.4
Metatarsus	BTl	19	19.39	0.04	14	19.8	0.05	16	18.1	0.05	− 1.9	7.1	9.0
Metatarsus	BTm	19	19.92	0.04	14	20.3	0.05	16	17.9	0.04	− 2.0	10.4	12.4
Metatarsus	CD	19	7.43	0.07	14	7.1	0.07	16	6.0	0.06	5.1	21.2	16.2
Metatarsus	DFp	19	22.50	0.04	14	22.6	0.04	16	20.7	0.04	− 0.2	8.2	8.4
Metatarsus	Dp	19	35.47	0.04	14	35.2	0.05	16	31.5	0.04	0.8	11.9	11.1
Metatarsus	DVl	19	23.46	0.03	14	23.0	0.02	16	21.8	0.05	1.8	7.5	5.7
Metatarsus	DVm	19	24.53	0.04	14	24.0	0.04	16	21.8	0.04	2.0	12.0	10.0
Metatarsus	GCD	19	11.17	0.09	14	10.9	0.11	16	8.7	0.05	2.9	25.5	22.6
Metatarsus	GDD	19	39.62	0.09	14	38.6	0.09	16	30.9	0.06	2.7	24.6	21.9
Metatarsus	GL	19	27.52	0.03	14	27.4	0.02	16	25.1	0.03	0.5	9.2	8.7
Metatarsus	PL	19	26.90	0.03	14	27.2	0.03	16	24.8	0.03	− 1.3	8.3	9.5
Metatarsus	SD	19	22.59	0.10	14	21.1	0.09	16	17.7	0.09	6.8	24.3	17.5
Metatarsus	SDD	19	19.37	0.05	14	19.0	0.07	16	16.2	0.07	2.1	17.9	15.9
Pelvis	DAm	1	11.45	#N/A	2	9.6	0.34	9	7.3	0.4	17.8	44.5	27.2
Pelvis	DPmin	1	#N/A	#N/A	2	7.5	0.28	9	7.1	0.13	#N/A	#N/A	5.9
Pelvis	DPS	1	19.14	#N/A	2	16.1	0.29	9	12.7	0.29	17.4	40.5	23.4
Pelvis	GBA	1	13.90	#N/A	2	13.8	#N/A	9	12.4	0.03	0.7	11.4	10.7
Pelvis	GBTc	1	24.00	#N/A	2	21.0	#N/A	9	38.6	0.7	13.3	− 46.6	− 59.1
Pelvis	GBTi	1	13.60	#N/A	2	11.7	#N/A	9	11.1	0.03	15.0	19.9	5.0
Pelvis	GL	1	31.40	#N/A	2	29.2	#N/A	9	27.0	0.04	7.3	15.2	8.0
Pelvis	LA	1	44.38	#N/A	2	46.1	0.00	9	41.4	0.02	− 3.8	7.0	10.8
Pelvis	LAR	1	31.08	#N/A	2	34.6	0.10	9	30.7	0.08	− 10.7	1.2	11.9
Pelvis	LFo	1	67.17	#N/A	2	66.7	0.05	9	62.0	0.05	0.8	8.1	7.3
Pelvis	LS	1	102.20	#N/A	2	100.0	0.04	9	90.4	0.06	2.2	12.3	10.1
Pelvis	SB	1	14.33	#N/A	2	12.2	0.19	9	12.2	0.06	16.1	16.3	0.2
Pelvis	SBl	1	92.40	#N/A	2	83.7	#N/A	9	81.5	0.03	9.9	12.6	2.7
Pelvis	SC	1	6.80	#N/A	2	6.1	0.05	9	5.6	0.05	10.9	19.0	8.2
Pelvis	SH	1	27.54	#N/A	2	24.4	0.00	9	22.1	0.05	12.1	21.9	9.8

#### Percent difference between the groups

For the identification of castrates versus full males, the measurements showing the clearest separation come from the radioulna and, to a lesser degree, the humerus, femur, and metatarsus. From our measurements, the tibia is the most unaffected by castration, and the metacarpus is the second least affected.

Regarding the humerus, the proximal breadth (Bp) and the depth of the caput (DC) show the clearest distinction (3.6% and 3.7%, respectively), and the height of the trochlea (HT) and smallest breadth of the diaphysis (SD) to a lesser degree. All these measurements have shown high resistance to measurement error, falling below 1.25%.

For the radioulna, the greatest breadth of the proximal articular facet (BFp), the smallest circumference of the diaphysis (CD), depth of the distal end (Dd), physiological length (PL), smallest breadth of the diaphysis (SD), the greatest length of the radioulna (GL), smallest depth of the diaphysis (SDD), length of the olecranon (LO), and smallest depth of the olecranon (SDO) show the greatest separation. Of these measurements, the Dd and SDD approach the 2.5% threshold of measurement error, but the amount of separation between the castrates and full males for these measurements (4.6% and 7.5%, respectively) surpasses this easily.

In the metacarpus, the clearest separation is in the measurements of the greatest breadth of the proximal articular surface (BAp) and smallest breadth of the diaphysis (SD). Of these measurements, the BAp is greatly affected by measurement error, which is slightly over 2.5%. At the same time, the SD is only slightly affected and thus the better candidate for a more accurate distinction.

For the femur, the smallest circumference of the diaphysis (CD) and the smallest breadth of the diaphysis (SD) show the greatest distinctions (4% and 3.4%, respectively). Both measurements are slightly affected by measurement error, below 1.25%.

The metatarsus shows the best distinction in the smallest circumference of the diaphysis (CD) and smallest breadth of the diaphysis (SD) (5.1% and 6.8% respectively) and minor distinction in the greatest circumference of the diaphysis (GCD) and the greatest depth of the diaphysis (GDD). The SD and GCD fall slightly over the 2.5% threshold, while the GDD greatly surpasses it, but the CD stays well below, so from the metatarsus measurements, the CD seems the most reliable.

The table and figure show that for most measurements, castrates are slightly smaller in bone size than full males, in all dimensions, with several exceptions only in the lower limb bones, i.e., metacarpus and metatarsus. The metatarsus is the largest bone, and the radioulna is the smallest bone for castrated males compared to full males. The greatest separation is primarily found in breadth, depth, and circumference measurements. However, it is not found in the length measurements, such as reported for other species. Our results only partly align with other osteometric studies that demonstrate that castrates have slimmer and lengthier limb bones compared to full males due to a delay in epiphyseal fusion (e.g., Hobday 1914; Silberberg and Silberberg 1971; Davis 2000; Telldahl et al. 2012).

For reindeer the picture appears slightly more complex. Castrated reindeer is slightly smaller in all bone measurements (except for some metapodial measurements), and their bones are thus marginally slimmer and shorter than full males. Nonetheless, length is not primarily affected, and the only three length measurements that are affected to a greater extent are found in the radioulna. The rest of the length measurements for castrates are negligibly smaller than for males, hovering around a 1% difference, which means that in most cases, the measurement error is greater than the measured length differences between the two groups.

For the separation of females from full males, all measurements show a separation greater than 8% (except for the metatarsal depth of the lateral verticulus (DVl) measurement and measurements of the pelvis), with most measurements falling between 10 and 20% size difference and few measurements above that. All measurements show that females are smaller and easily separated from full males, which for *Rangifer tarandus tarandus* has already been demonstrated by Puputti and Niskanen (2009). This offers good prospects for archaeological application. Our results show the biggest size differences between females and full males are in the relative depth and circumference measurements, with all % differences above 20% pertaining to these two dimensions. The metapodials are least affected by growth in length (around 8–9%), while other bones are affected slightly more by growth in this dimension (12–15%), and only the length of the olecranon of the radioulna (LO) being considerably affected (20% difference). The % difference between females and males in the pelvis is generally high. Still, we cannot make any robust statements because of the small sample size.

The separation between females and castrates is almost as clear-cut, with only the smallest depth of the diaphysis of the radioulna (SDD) being practically inseparable between the two groups. All % difference results of the measurements from female bones show that in all respects, female bones are by far the smallest of the three groups (except the SDD), with most measurements falling between 10 and 20% difference.

#### Covariants of variation

The CV characterizes the amount of variation and allows a comparison of variability of different measurements of separate bone elements among different groups (Yablokov 1974, p 8). Covariants of variation could be due to different populations, environmental and genetic factors, individual properties of the animals, and observer error in measurements. Yablokov (1974, Table 28) compiled a table on the variability of body measurements for mammals, which suggests an average variability between 3 and 5 for linear measurements of the post-cranial skeleton. The CVs in our study can indicate the effect of castration on morphological heterogeneity.

A general rule of using the CV is that smaller populations or sample sizes generally yield lesser variability (Haldane 1955, in Yablokov 1975). This can be expected then, too, for our studied samples, as full males and castrates generally have the smallest and females the largest sample sizes in our sample. Furthermore, if osteological studies on the CV of other species are compared, the general pattern emerges that CVs are higher if samples are included from different breeds, flocks, or subpopulations (e.g., for sheep, see, Guintard and Lallemand 2003; Clutton-Brock et al. 1990). Our reindeer samples come from several different subpopulations (see Materials); thus, we expect our CVs to be slightly higher than between 3 and 6.

Most of the CVs of our studied samples lie between 3 and 6, with exceptions both above and below. An apparent exception in our study is the CV of the measurements from the pelvis bone, which are exceptionally high and reach up to 66 in one case. Studies on other animals have also reported high CVs of pelvis measurements. These high values are probably in part because the pelvis bone keeps growing throughout an animal’s life (Hufthammer 1995; Popkin et al. 2012; Takken Beijersbergen and Hufthammer 2012), and in part due to measurement error (the “Measurement error” section; Popkin et al. 2012).

The notion that lower CVs might be expected from smaller samples (for us, the castrate and full male groups) does not apply to our results. We recognize that the small sample effect on lowering the CVs might not apply fully to this study partly because our sample size differences are not that big.

The variation in full males is slightly higher overall than in the other two groups, followed by castrates and the females, with exceptions. Females show relative homogeneity (average CV of 4.0, excluding pelvis) in comparison to full males (4.7) and castrates (4.5). We think the raised levels of variability in full males, despite the small sample size and the factors influencing variability mentioned above, could be related to stress during the rutting and associated lack of food during this period. Overall, greater intrasex phenotypic variability has been reported for males in several studied species, including humans (Lehre et al. 2009; Popkin et al. 2012). The low variation in females and the higher variation in full males and castrates can also be seen in, for example, our simple variable combinations (the “Mennerich’s indices and simple variable combinations” section).

A likely tempering factor in the CVs of the castrated group is that castrated reindeer are not subject to the male hormonal shifts and do not exhaust or stress themselves during the rut, nor do they limit their food intake during this time. Aggravating effects of variability in castrates (over females) could be due to two additional factors: age and severity of castration which both affect bone growth.

Overall, the greatest variation in castrated and full male reindeer bones can be found in the measurements of circumference, depth, and breadth with variables related to the diaphysis. The effect of greatest variation in smallest breadth (SD) measurements was also perceived by Guintard and Lallemand (2003) in their study on sheep metapodials. The breadth and depth measurements show the most variation after the diaphysis variables, while the length measurements show the least variation. This holds true for female reindeer individuals to a lesser extent: for females, several variables for depth, breadth, and length also score high on CV, while several measurements for breadth and depth also score low on CV.

That the measurements related to the diaphysis have high CV and variables related to the longitudinal axis have low CV values could be explained by the diaphysis measurements’ relatively higher degree of inter-observer error on the diaphysis variables. Another reason could be that there is less selective pressure on the morphology of the diaphysis in reindeer. In contrast, most selective pressure for males is exercised on the bone length and, thus, the longitudinal axis. This could have evolutionary roots in higher mating success for males of greater vertical sizes in domestic reindeer populations. This could perhaps be brought about through selection by reindeer herders for sizable males for breeding purposes or because taller males have greater success in mating for reasons relating to natural competition and survival rate, or a combination of both factors. That there is less order in the variation of female reindeer bones could be related to less selective mating or survival pressure for the body size and shape of females, brought about by herders or natural circumstances.

### Mennerich’s indices and simple variable combinations

**Fig. 11:**
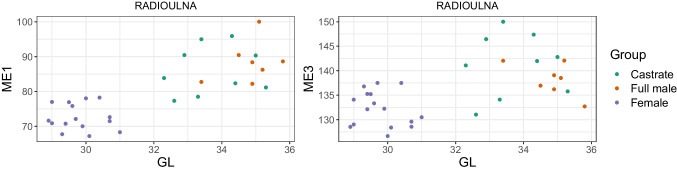
Plot of Mennerich’s index 1 (left) and 3 (right) for complete radioulna plotted against the greatest length (GL)

**Fig. 12:**
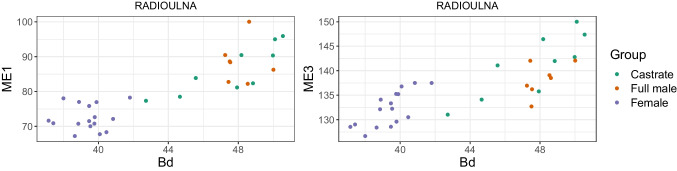
Plot of Mennerich’s index 1 (left) and 3 (right) for complete radioulna plotted against the distal breadth (Bd)

Combining length variables with depth and breadth variables to understand bone gracility between sexes, breeds, and subspecies is common practice in zooarchaeological osteometric studies (e.g., Boessneck et al. 1964; Guintard 1996; Guintard and Lallemand 2003; Telldahl et al. 2012). Here, we employed the gracility indices of Mennerich (1968). The separation of the three groups in scatter plots using Mennerich’s indices 1 and 3 mostly give good results for dividing females from the two male groups for all elements, but generally weak results for the division between full males and castrated reindeer (Figs. 11 and 12). The figures display the elements’ slenderness, i.e., robusticity, in relation to the greatest length (GL) and distal breadth (Bd). Here, we only present our best results of group separation. Still, all other scatterplots of Mennerich’s indices 1 and 3 and their combinations in one scatterplot can be viewed in our GitHub repository, in which slenderness is also presented in relation to the smallest breadth of the diaphysis (SD).

Females are always well separated from the male groups in all elements, except in Mennerich’s Indices 1 and 2 plotted in relation to the SD of the metatarsus. For castrates, the results on the radioulna suggest a continuity in slenderness between females, castrates, and males, though also that most castrates match males in slenderness and provide moderate separation. Index 1 (which includes the SD and GL) and 3 (which includes the Bd and GL) plotted against the GL produce two size clusters each. At the same time, the castrate samples scatter the full male, more concentrated cluster in relation to the GL and the ratios of the Bd (index 1) and SD (index 3) to the GL. Indices 1 and 3 plotted against the Bd produce one elongated size cluster each. Both indices for the radioulna somewhat resemble the sex and castration-related slenderness distributions that we see in other species (e.g., *Bos taurus* in Telldahl et al. 2012), i.e., castrates fall in size and slenderness between males and females. This effect for indices 1 and 3 is more pronounced when plotted in relation to the Bd. For radioulna, the spread of the castrates is greater than the spread of full males, i.e., castrates encompass the ranges of full males and beyond. We did not get clear visual separations between the groups when combining Mennerich’s indices 1 and 3 in one scatter plot.

**Fig. 13:**
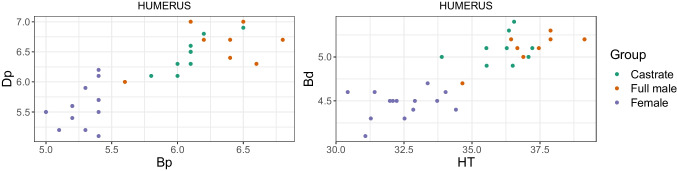
Plot of humerus proximal variables proximal depth (DP) and proximal breadth (Bp) (left), and the distal variables distal breadth (Bd) and trochlear height (HT) (right)

**Fig. 14:**
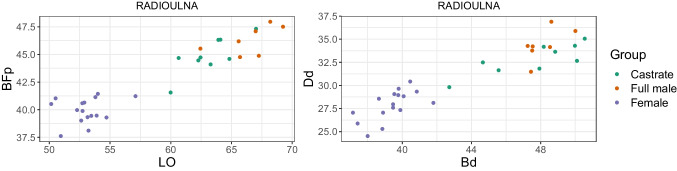
Plot of the radioulnar proximal breadth of the proximal articular facet (BFp) and length of the olecranon (LO) (left), and the distal variables distal depth (Dd) and distal breadth (Bd) (right)

**Fig. 15:**
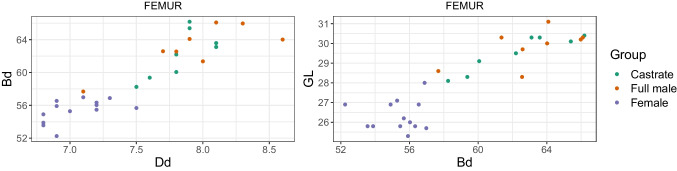
Plot of femur distal breadth (Bd) and distal depth (Dd) (left), and the combination of the greatest length (GL) and distal breadth (Bd) for complete bones (right)

**Fig. 16:**
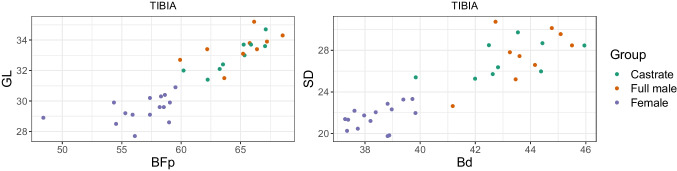
Plot of the greatest length (GL) and breadth of the proximal articular facet (BFp) (left) and the combination of the breadth of the diaphysis (SD) and distal breadth (Bd) for complete tibia bones (right)

**Fig. 17:**
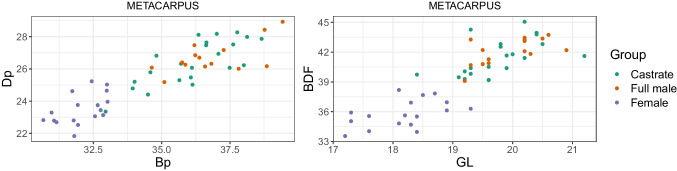
Plot of the proximal metacarpal proximal depth (Dp) and proximal breadth (Bp) (left), and the combination of the breadth of distal fusion line (BDF) and the greatest length (GL) of complete bones (right)

**Fig. 18:**
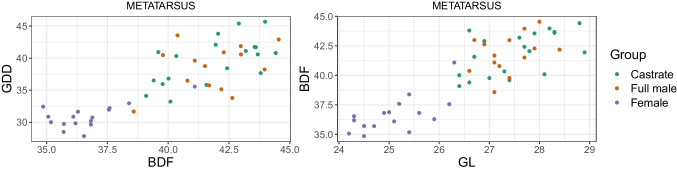
Plot of the metatarsal greatest depth of the diaphysis (GDD) and breadth of distal fusion line (BDF) (left) and the BDF in combination with the greatest length (GL) (right) for complete metatarsal bones

**Fig. 19:**
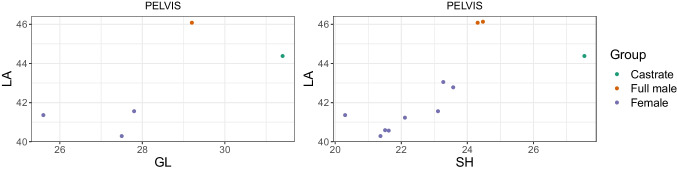
Plot of pelvis length of the acetabulum including lip (LA) in combination with greatest length (GL) (left) and in combination with the smallest height of the shaft ilium (SH) (right)

Two variables are combined in the simple variable combinations to produce a scatter plot. Here, we focus only on two variable combinations per element (Figs. 13, 14, 15, 16, 17, 18, and 19). The complete body of scatterplots from the variable combination analysis can be viewed in our GitHub repository. Overall, none of the variable combinations produced clear-cut group separation. Still, many produced elongated scatter plot patterns characteristic of three-group plots in which castrates and full males overlap in slenderness but also in which castrates lie between females and full males in gracility.

The radioulna performed best overall and is, therefore, most suitable for identifying castrates. Several variable combinations are suitable for separate proximal and distal parts and complete bones. The females are easily separated from the full males, and the castrates usually occupy the “space” between females and full males with overlap with the full males (Fig. 13). The humerus (Fig. 14) also displayed a clear elongated pattern good for discerning castrates, albeit less clear than the radioulna. This element shows a clear separation between females and full males, with castrates occupying the space between females and males and overlapping with the latter category. We found the femur less helpful, and the tibia gave the least clear scatter plot elongation characteristics from the upper long bones. However, some variable combinations still display castrates occupying a position between females and full males (Figs. 15 and 16). For the upper long bones, there is one male outlier: an unusually small individual with several variables in the female range.

The metapodials showed the lowest suitability. Nevertheless, some of the metapodial variable combinations still showed elongated size clusters (Figs. 17 and 18), with several measurements from castrates falling in between females and males in general, though displaying more overlap with full males than the other elements. There is one exceptionally large female that only presents itself in the metapodial measurements.

The pelvis could have great potential for three-group separation due to its hormone-related growth and late fusion time. However, the difficulty with the pelvis lies in its late fusion time: we had to omit many measurements because parts of the pelvis were not fully fused or fusing. This resulted in an even smaller sample size. Thus, our variable combination scatterplots of the pelvis could give indications of measurements that might work well together but will need future investigation with a more comprehensive modern reindeer sample. Several pelvis measurements (combinations) seem to show potential for three-group separation in reindeer (Fig. 19).

### Measurement correlation and linear discriminant analysis

All measurements are highly correlated to each other (Fig. SI1 to SI6 in the Supplementary Information). This stresses a crucial feature of the long bones of domestic reindeer, namely, that they display a great uniformity in proportion and design. The axis along which the measurements were taken is an important factor that rules the correlation. For example, the length measurements (GL, LI, GLl, GLC, PL, and LO) all have a correlation to each other of 0.94 to 1. Also, the depth measurements are highly correlated (all above 0.82 except for the metapodials, which are all above 0.72). The breadth measurements are also highly correlated but to a lesser extent with some exceptions in the femur breadth measurements and the smallest breadth (SD) in the metapodials. High correlations between measurements mean that for our first variable reduction for the pLDA models, it would likely not matter much which variables are chosen for similar model performance, as long as they are from a different axis.

For both the humerus and the tibia, all measurements display high levels of correlation inside the element. Also, the radioulna and femur show major homogeneity with exceptions only in the humerus’s BT (breadth trochlea) and the SDD (smallest depth diaphysis) of the radioulna. Variables of the metapodials show a lesser uniformity in general. The lower correlations of the PL (physiological length), DFp (depth fusion proximal), and BA (breadth between articular crests) are most pronounced in the metacarpus. In contrast, the Bp (breadth proximal), PL, and BA show the least correlation in the metatarsus.

Here, we show the results of our three penalized LDA models: (1) between the three groups, (2) between castrates and full males, and (3) between castrates and full males + females. We used our study on the correlation between the variables to reduce our total amount of variables per element to subsets of variables usable for discriminant analysis (see the “Variable correlations, multicollinearity, and variable reduction” section). Furthermore, we divided our variables into subsets of variables of complete bones and separate proximal and distal bone parts to aid future application to fragmented archaeological reindeer bone assemblages (see the “Variable correlations, multicollinearity, and variable reduction” section). The two most important variables per element and element part for our models are highlighted in the results tables.

For our three-group model (Table 4), all our results are statistically significant (*p* < 0.05), especially the humerus, radioulna, and metacarpus score exceptionally well. This means that the front limb bones are most diagnostic for the purpose of distinguishing between the three groups. Females are always correctly classified between 95 and 100% of the time. In all cases, the length measurements are seen as the most important in the model for the classification of complete bones, and both breadth and depth measurements are the second most important classifier in the model. The breadth is the most important for separate distal and proximal ends. Complete, distal, and proximal parts score nearly as well as complete bones in this model.

**Table 4:** Discriminant analysis classifying sex and castration status based on three-group (castrate, full male, female) analysis for complete bones and proximal and distal bone parts separately. The two most important variables identified by the model for metric three-group separation are in bold

Element	Part	Measurements	Accuracy	F1-score			Balanced accuracy			*p*-value
				Female	Castrate	Male	Female	Castrate	Male	
Humerus	Complete	**GLC**, **Bp**, SD, BT, HT, DC, Dp, Dd	80.6	96.6	66.7	66.7	97.1	76.5	76.9	0.0001
Humerus	Proximal	**Bp**, **DC**, Dp	83.9	96.6	75	70.6	97.1	81.1	81	0
Humerus	Distal	**Bd**, **BT**, HT, HTC, Dd	83.9	100	70.6	70.6	100	78.8	81	0
Radioulna	Complete	**PL**, **Bp**, Bd, Dd, Dp, LO, SDO	96.9	100	94.7	92.3	100	97.8	92.9	0
Radioulna	Proximal	**Bp**, **BFp**, Dp, LO, SDO	96.9	100	94.7	92.3	100	97.8	92.9	0
Radioulna	Distal	**Bd**, **Dd**	84.4	97	70.6	71.4	96.9	79	81.7	0.0001
Metacarpus	Complete	**GL**, **Bp**, Dp, SD, Bd, BDF, BA	84.9	100	84	63.6	100	86.4	73.7	0
Metacarpus	Proximal	**Bp**, **Dp**, BAp, DFp	77.4	97.1	76	47.6	98.6	78.7	65.3	0
Metacarpus	Distal	**Bd**, **BTm**, BTl, DVm, DVl, BDF, BA, SDD	81.1	97.1	78.3	64	98.6	81.2	74.7	0
Femur	Complete	**GLC**, **Bp**, DC, SD, Bd, Dd, BT	79.3	96.3	66.7	62.5	96.9	76.5	74.1	0.0002
Femur	Proximal	**Bp**, **DC**	75.9	96.3	57.1	58.8	96.9	70.2	71.7	0.0007
Femur	Distal	**Bd**, **Dd**, BT	86.2	96.3	80	75	96.9	85.1	82.7	0
Tibia	Complete	**PL**, **SD**, Dd, Dp, BFp, BFd	78.8	100	58.8	63.2	100	71.5	75	0.0001
Tibia	Proximal	**BFp**, **Dp**	72.7	90.9	42.9	63.2	91.7	62.5	75	0.0014
Tibia	Distal	**Bd**, **Dd**, BFd	75.8	93.8	53.3	63.2	94.4	68.1	75	0.0004
Metatarsus	Complete	**GL**, **Bp**, Dp, SD, Bd, BDF, BA	83.7	96.8	82.9	69.2	96.9	86.4	77.9	0
Metatarsus	Proximal	**Bp**, **Dp**, BAp, DFp	67.3	90.9	68.2	28.6	93.8	72.8	55	0
Metatarsus	Distal	**Bd**, **BTm**, BTl, DVm, DVl, BDF, BA, SDD	79.6	93.8	75.7	69	95.4	80.2	78.6	0

Overall, the model works well in identifying females from males and castrates. Castrates and males are significantly more difficult to classify correctly. The F1-score range for females is [90, 9–100], whereas, it is [42, 9–94, 7] for castrates and [28, 6–92, 3] for males, which suggests major variation between the performance of the model between different bone elements or elemental parts.

In our two-grouped models of castrates versus full males (Table 5), we can see a clear difference in the model performance compared to the previous models. Statistically, significant models for this set of two-grouped models are the proximal and complete humerus, the proximal and complete radioulna, the complete metacarpus, the complete metatarsus, and the distal femur. These elements and their parts all show F1-scores of 75% or above. Especially complete bones are useful for separating full males from castrates, but separate epiphyseal ends can also be used successfully. It is again evident that the length and breadth measurements are most important in classifying the complete bones. The breadth measurements are most important in classifying the distal and proximal ends.

**Table 5:** Discriminant analysis classification based on two-group (castrate, full male) analysis for complete bones and proximal and distal bone parts separately. The two most important variables identified by the model for metric separation of the two groups are in bold

Element	Part	Measurements	Accuracy	F1-score	Balanced accuracy	*p*-value
			Castrates v males	Castrates v males	Castrates v males	
Humerus	Complete	**GLC**, **Bp**, SD, BT, HT, DC, Dp, Dd	76.5	77.8	76.4	0.0421
Humerus	Proximal	**Bp**, **DC**, Dp	76.5	77.8	76.4	0.0421
Humerus	Distal	**Bd**, **BT**, HT, HTC, Dd	58.8	58.8	59	0.4063
Radioulna	Complete	**PL**, **Bp**, Bd, Dd, Dp, LO, SDO	93.8	94.7	92.9	0.0014
Radioulna	Proximal	**Bp**, **BFp**, Dp, LO, SDO	87.5	88.9	87.3	0.0086
Radioulna	Distal	**Bd**, **Dd**	75	75	76.2	0.102
Metacarpus	Complete	**GL**, **Bp**, Dp, SD, Bd, BDF, BA	83.3	87.5	79.9	0.0036
Metacarpus	Proximal	**Bp**, **Dp**, BAp, DFp	66.7	76.9	59.7	0.3077
Metacarpus	Distal	**Bd**, **BTm**, BTl, DVm, DVl, BDF, BA, SDD	72.2	78.3	69.5	0.1143
Femur	Complete	**GLC**, **Bp**, DC, SD, Bd, Dd, BT	68.8	70.6	68.8	0.1051
Femur	Proximal	**Bp**, **DC**	56.2	53.3	56.2	0.4018
Femur	Distal	**Bd**, BT, **Dd**	75	75	75	0.0384
Tibia	Complete	**PL**, **SD**, Dd, Dp, BFp, BFd	61.1	66.7	61.1	0.2403
Tibia	Proximal	**BFp**, **Dp**	61.1	63.2	61.1	0.2403
Tibia	Distal	**Bd**, **Dd**, BFd	66.7	66.7	66.7	0.1189
Metatarsus	Complete	**GL**, **Bp**, Dp, SD, Bd, BDF, BA	78.8	82.1	77.8	0.0091
Metatarsus	Proximal	**Bp**, **Dp**, BAp, DFp	57.6	69.6	52.8	0.5731
Metatarsus	Distal	**Bd**, **BTm**, BTl, DVm, DVl, BDF, BA, SDD	69.7	73.7	69	0.1076

Our last pLDA model (Table 6) shows how effectively the castrates are separated from full males and females if the full males and females are grouped together. Only the models of the complete metapodials and complete radioulna are significant (*p* =  < 0,05). These elements and their elemental parts give good overall accuracy for correctly classifying castrated reindeer. Again, the length and breadth measurements seem to be the most important for classifying complete bones. The breadth measurements, and to some extent depth measurements, are the most important for classifying proximal and distal parts. Considering both the accuracies and *p*-values, this model shows that classifying castrates if females and full males are grouped together has limited (practical) use.

**Table 6:** Discriminant analysis classification based on two-group (castrate, full males + females) analysis for complete bones and proximal and distal bone parts separately. The two most important variables identified by the model for separating the groups are in bold

Element	Part	Measurements	Accuracy	F1-score	Balanced accuracy	*p*-value
			Castrates v males + females	Castrates v males + females	Castrates v males + females	
Humerus	Complete	**GLC**, **Bp**, SD, BT, HT, DC, Dp, Dd	77.4	58.8	71	0.283
Humerus	Proximal	**Bp**, **DC**, Dp	64.5	26.7	52	0.8391
Humerus	Distal	**Bd**, **BT**, HT, HTC, Dd	80.6	62.5	73.2	0.1613
Radioulna	Complete	**PL**, **Bp**, Bd, Dd, Dp, LO, SDO	84.4	70.6	79	0.0792
Radioulna	Proximal	**Bp**, **BFp**, Dp, LO, SDO	75	50	65.7	0,4334
Radioulna	Distal	**Bd**, **Dd**	81.2	66.7	76.8	0.1629
Metacarpus	Complete	**GL**, **Bp**, Dp, SD, Bd, BDF, BA	71.7	65.1	70.5	0.033
Metacarpus	Proximal	**Bp**, **Dp**, BAp, DFp	66	57.1	64.4	0.1647
Metacarpus	Distal	**Bd**, **BTm**, BTl, DVm, DVl, BDF, BA, SDD	83	80.9	83.5	0.0001
Femur	Complete	**GLC**, **Bp**, DC, SD, Bd, Dd, BT	69	30.8	55.4	0.7394
Femur	Proximal	**Bp**, **DC**	62.1	26.7	50.6	0.9234
Femur	Distal	**Bd**, BT, **Dd**	65.5	28.6	53	0.8504
Tibia	Complete	**PL**, **SD**, Dd, Dp, BFp, BFd	66.7	35.3	56.2	0.8364
Tibia	Proximal	**BFp**, **Dp**	63.6	25	50.7	0.9112
Tibia	Distal	**Bd**, **Dd**, BFd	66.7	26.7	52.8	0.8364
Metatarsus	Complete	**GL**, **Bp**, Dp, SD, Bd, BDF, BA	79.6	73.7	78.5	0.0049
Metatarsus	Proximal	**Bp**, **Dp**, BAp, DFp	73.5	66.7	72.5	0.0509
Metatarsus	Distal	**Bd**, **BTm**, BTl, DVm, DVl, BDF, BA, SDD	85.7	80	83.5	0.0002

## Discussion and conclusion

Our research demonstrates that castration considerably affects bone size and shape. It can be induced that this is (at least partially) due to the effect of castration on epiphyseal fusion time and, thus, bone growth. Our methods are only appropriate for detecting castration if the reindeer is castrated before epiphyseal fusion is completed. Different elements might be more or less affected in the case of earlier or later castration ages. In conclusion, about how castration presents itself in reindeer bones, it can be said that most bones exhibit both full male and female features after castration of the reindeer; some measurements appear full male-like, while other osteometric measurements of the same element appear more feminine. Our results are also relevant beyond castration because it is the most comprehensive study to date on the osteometric size differences between full male and female reindeer.

Our results show that the metapodials are least affected by castration and are least suitable for detecting castration in the archaeological record. In contrast, other limb bones are affected considerably more by castration. A logical explanation of this phenomenon lies in the fusion time of the separate osteological elements. Metapodials are early fusing, that is to say, between 18 and 30 months of age, while the other limb bone elements fuse substantially later (Hufthammer 1995; Takken Beijersbergen and Hufthammer 2012). For example, the femur fuses between 36 and 48 months, as do the distal radioulna and proximal tibia, while the proximal radioulna and proximal humerus fuse even later (between 42–48 months and 42–54 months, respectively). The distal humerus and proximal radius fuse relatively early, between 6–15 months and 4–10 months of age, respectively (Hufthammer 1995; Takken Beijersbergen and Hufthammer 2012).

As the hormonal changes due to castration and its effect on epiphyseal fusion can only come into effect after castration, it is only rational that (mostly) the later fusing elements are affected by it. This means that our method will not detect reindeer castrated after epiphyseal fusion is completed in the archaeological record. Reindeer castrated before most bones have finished fusion might be easier to detect.

Our research also shows that the effect of castration does not necessarily ensure an elongation of the long bones, as suggested by several other studies (Hobday 1914; Silberberg and Silberberg 1971; Davis 2000). Some of the castrated reindeer in our sample show greater length measurements in different elements in several cases, but most do not. Castrated reindeer bones are in our study primarily linked to larger sizes in terms of length, breadth, and depth relative to females, and both overlapping and smaller sizes in the same dimensions relative to full males.

Regarding bone shape, our use of Mennerich’s indices 1 and 3 also suggest that castrates usually do not exhibit an elongated shape in comparison to females and full males, but that their bone shape falls mostly between females and full males and has great overlap with full males in terms of elongational shape. An exception is Mennerich’s index 3 performed on the radioulna plotted against the radioulnar Bd and GL, which shows a clear elongation in both cases, but again with great overlap with full males and many of the individuals leaning towards female proportions.

Castrated reindeer have lower heterogeneity in measurements of the anterior–posterior and medial–lateral axis compared to full males and higher heterogeneity than females on these axes. Measurements of the longitudinal axis show approximately the same homogeneity as in full males and females. The combined CV, bone shape, and bone length results show that the long bones are relatively stable in the longitudinal dimension. Castration enhances heterogeneity in the male group (comprising both full males and castrates) but mitigates heterogeneity when the two male groups are considered separately.

The osteological plasticity in the cranial-caudal and medial–lateral dimensions suggests external influence over these measures. These could be linked to environmental conditions or lifestyle (Weinstock 1997; Niinimäki and Salmi 2016; Pelletier et al. 2020) and body mass (Puputti and Niskanen 2008).

Individual elements might not be easily identified to castration status. Still, if the sample size is sufficiently large, then the presence of three groups, and thus castration, is presented as an elongated or trimodal distribution. Bimodal distributions indicate female and full male groups. Castrates may be detected through Mennerich’s indices on the radioulna discussed above and through a combination of different variables presented in scatterplots. The best metric separations were achieved in particular by the radioulna with, for example, BFp × LO (proximal bone fragments) and Dd × Bd (distal bone fragments), the humerus Dp × Bp (proximal bone fragments), and Bd × HT (distal bone fragments), and; the femur Bd × Dd (distal bone fragments) and GL × Bd (complete bone finds) and other measurement combinations (see our GitHub repository) are suited for this purpose. The LA, in combination with the GL and SH measurements of the pelvis, also shows potential but should be tested in future research due to our limited sample size.

Linear discriminant analysis may also be used for group differentiation and has, in our study, shown useful for separate proximal and distal parts and complete bones. The results of our pLDA models for the three different group arrangements (separating all three groups from each other; separating castrates from full males; separating castrates from both full males and females, if full males and females are grouped together) suggest that separating castrated reindeer bones from male and female bones is most effective when all three groups are considered separately, as the pLDA models of our first group arrangement performs best. The reason for this increased accuracy over the other two arrangements is most likely that castrates are more easily separated from females than full males. Thus, the correct castrate-female classifications boost the models’ performance. Our second group arrangement, which aims to separate only full males and castrates, performs very well for several complete bones and bone parts like the proximal and complete humerus and radioulna, complete metapodials, and distal femur. Our third and last group arrangement only has acceptable accuracies and p-values for complete metapodials and radioulna. For most models, the length and breadth measurements were most important for group separation, and overall, the radioulnar element performed best in all arrangements.

The application of these models could thus be useful for studying fragmented assemblages containing proximal humerus, radioulna, and distal femur. The complete bones are also successful in correctly classifying castrated versus full male bones, and thus our model could be applied where entire elements or skeletons are found. Usually, archaeological excavations in Fennoscandia yield mostly fragmented reindeer bones due to the practice of marrow extraction (Harlin et al. 2019), but complete bones are found as well in, for example, draft reindeer burials (Collinder 1949, p 136; Spencer 1978, p 69; Roué 2012, p 50).

Castrates showing bigger or smaller bone sizes might have, besides castration age-related effects, been subject to different strengths of castration, nutritional plane, come from a different reindeer population, or might have been selected for castration based on individual physical or mental properties. This, together with the nature of the domestication process, might have confounding influences on the archaeological application of our methods and furthermore presents limitations in our study (sample).

Firstly, it is known that nutrition affects the time of fusion and bone growth, though the effects are not equal among all skeletal elements or element components. In several studied mammal species, low-nutrition individuals have smaller bones than high-nutrition individuals in general (Popkin et al. 2012). Nutrition has been shown to affect bone growth considerably and body weight in reindeer likewise (Thomas and Everson 1982; Klein et al. 1987; Helle and Kojola 1994; Kuzyk et al. 1999). Our studied reindeer bone sample included reindeer populations from different geographical origins from separate years and different lifestyles. These separate populations are bound to have experienced different nutritional resources and resource availability. Unfortunately, we could not take this into account due to limited sample availability and missing information on the nutritional plane of our reindeer individuals. Furthermore, differences between the nutritional plane of castrated reindeer and other (untrained) reindeer of the same herd in ancient reindeer populations will influence their archaeological detection likewise.

Secondly, our sample contains reindeer individuals from different lifestyles: free-ranging, captive, and working reindeer (racing and pulling). These different lifestyles can induce distinctive stress changes in the bones so that their shape can be affected (Niinimäki and Salmi 2016; Pelletier et al. 2020). Pelletier et al. (2020) have found evidence that captive individuals among domestic reindeer did not seem smaller compared to free-ranging individuals. However, they also found that working reindeer tended to be slightly larger than free-ranging reindeer. This could be explained by the fact that working reindeer were often selected for their physical properties (Bosi 1960, p 114; Paine 1994, pp 25–28; Pelletier et al. 2020), and/or because working reindeer were and are often supplementarily fed (Van den Berg 2022, unpublished manuscript). We decided not to include lifestyle in our analysis as this would reduce our sample size per group to insufficient quantity for statistical analysis. Besides, lifestyle status was unknown for many individuals from our castrated sample. Ancient reindeer individuals will most likely have been free-ranging or working animals; thus, size differences related to their lifestyle can be expected.

Thirdly, our reindeer bone samples come from different time periods, collected between 1869 and 2020, most of which are modern and stem from the second half of the last century to the present day. This could have two important implications for this study and its archaeological application. First of all, herders have reported that in the course of the twentieth century, domesticated reindeer have decreased in body size due to a multitude of factors (e.g., Pitkänen et al. 1994, pp 93–94). This means that our sample may contain reindeer of different sizes linked to the time period during which they were collected. These size differences might have had implications for the overlapping ranges between the groups and, thus, for all our results, possibly blurring the separation between them.

Fourthly, body size reduction is a common characteristic in domestic species compared to their wild counterparts (Tchernov and Horwitz 1991; Zeder 2006; Zeder and Hesse 2000). For reindeer specifically, recent archaeological bone analysis of twelfth–seventeenth-century Sámi offering sites suggests a size reduction between this past and modern domestic reindeer (Salmi et al. 2020a). This means that our analysis’s absolute sizes might not be relevant for castrate detection in (all) archaeological assemblages. However, the results of the relative size differences of different variables (and their combinations) between the groups are significant because they show which variables are influenced by castration and which are considerably less so. As follows, we recommend applying our methods on archaeological assemblages containing several or more reindeer individuals and not using them for the diagnostic purpose of single reindeer bone finds. When applied correctly, our methods can identify castrates in the archaeological record based on relative size between the three different groups.

Lastly, the strength of castration (the “Age, methods, and strengths of castration” section) might affect skeletal development as it allows gradations of emasculation (Skjenneberg and Slagsvold 1979, p 279; Paine 1994, pp 25–28). It is probable that the differences in bone development between the groups are directly due to hormone secretion and testosterone production in particular, which is known to cause the epiphysis to fuse with the diaphysis (e.g., Short 1980; Shahin et al. 1992). This leads us to think that more heavily castrated reindeer might be more affected by castration than reindeer that underwent a lighter form of castration. As we have no information on the castration strength from our castrated reindeer sample, we did not consider this in our analysis. Still, we expect that this variable possibly confounds our results and the application of our methods on any archaeological reindeer bone assemblage.

Despite our study’s limitations, we showed that castrated reindeer bones and bone fragments are identifiable through osteometric analysis in assemblages containing females, full males, and castrates. Additionally, due to differences in castration strength, our osteometric results of our castrates might have been more spread out and less centered; the same is expected for ancient reindeer individuals, so our study might provide a good frame of reference for the implication of castration strength for castrate bone sizes and shape relative to full males and females.

The highest accuracy for metric three-group separation was found for the front limb bones. This might be due to hormone-related growth patterns in especially the front limbs, that might be influenced by castration:

“*One does not want draft reindeer castrated too “severely.” If this occurs, the animal becomes lazy and is unwilling to work. Preferably they should be rather fast workers. It is most important that they do not exhaust themselves during the rutting season. It does not matter much if they still have a tendency to mate. They also retain more of the bull characteristics by this light form of castration; they remain heavy in the forequarters, which is an advantage when they are pulling*.” (Skjenneberg and Slagsvold 1979, p 281).

From the excerpt, we can deduce that the hormones that are influenced through castration have a perceivable effect on the front limb bones and that strength of castration leads to more feminine front limbs in live reindeer. As is apparent from our results, and which is thus conceivable, is that reindeer that were more “lightly” castrated may be harder to detect from osteological measurements while separating “severely” castrated, and therefore more feminine, reindeer from full males may be more straightforward.

Herding strategies are and have been extremely variable between ethnic reindeer herding groups in Fennoscandia and Siberia. The distinctive groups practicing reindeer herding today all have their own unique means of expression. The human-reindeer relationships of the North are by no means uniform (e.g., Jordan 2011; Mirov 1945). To all appearances, however, castration has always been an integral aspect of reindeer herding practices among the peoples of the circumpolar North. It has indeed been suggested that reindeer domestication might have started with the taming of castrated males (Ingold 1986; Bjørklund 2013). Therefore, as our data and analysis advance the understanding of reindeer skeletal development under the influence of castration, it offers opportunities to enrich our knowledge of ancient reindeer herding cultures through this method’s application on archaeological assemblages.

More interestingly, geldings may serve as evidence of a domesticating human-reindeer relationship before the genetic and phenotypic alteration of the domestic in relation to the wild reindeer herd takes place, indicating the removal of the species from the “wild” category. Beyond reindeer, this strategy may also prove fruitful for other domestic species. Eventually, after this method’s application on ancient bone assemblages, its results might help us reflect on prevailing definitions of domestication, management, taming, and human-animal domination.

Zooarchaeologists recognize the complexity of the environmental and biological influences under which bone growth and development are subject. Only through systematic study of these factors on skeletal development can we establish a thorough understanding of reindeer bone growth and biometry. A natural progression of this work is to analyze the influence of castration on the timing of epiphyseal fusion of reindeer (long) bones and how the osteological effects of castration are influenced by castration age and the reindeer’s lifestyle. New insights into osteo-environmental-hormonal interactions can guide us in our interpretations of archaeological bone assemblages and help us better comprehend ancient reindeer herding strategies. In turn, such insights can provide novel frameworks for, and new understandings of, contemporary human-reindeer encounters and relationships.

## Supplementary Information

Below is the link to the electronic supplementary material.

Supplementary file1 (DOCX 23 KB)

Supplementary file2 (PDF 8 KB)

Supplementary file3 (PDF 7 KB)

Supplementary file4 (PDF 10 KB)

Supplementary file5 (PDF 7 KB)

Supplementary file6 (PDF 7 KB)

Supplementary file7 (PDF 10 KB)

Supplementary file8 (PDF 8 KB)

## Data Availability

Code for statistical analysis is available upon request. Osteometric data becomes available after the end of the Domestication in Action project. All plot results of simple variable combinations and Mennerich’s indices 1 and 3 are available in our GitHub repository (https://github.com/mathildevandenberg/Castrated-Reindeer-Plots).

## Footnotes

1 Balanced Accuracy = (Sensitivity + Specificity) /2.

2 F1 = (Precision * Recall) / (Precision + Recall).

## Code availability

The code is available upon request.
