# Supplementary material for: The osteometric identification of castrated reindeer (Rangifer tarandus) and the significance of castration in tracing human-animal relationships in the North
Source: Archaeol Anthropol Sci. 2022 Dec 9;15(1):3. doi: 10.1007/s12520-022-01696-y (PMC9734228; doi:10.1007/s12520-022-01696-y)
Supplement: Supplementary file 1 — Supplementary file1 (DOCX 23 KB) [file 12520_2022_1696_MOESM1_ESM.docx]

**Supplementary Information**

Manuscript: What about Rudolph? The identification of castrated reindeer (*Rangifer tarandus*) bones and the significance of castration to trace human-animal relationships in the North.

Authors: Mathilde van den Berg, Henri Wallen, Anna-Kaisa Salmi

**Fig. SI1** The correlations between variables (measurements) taken from the humerus. For a definition of variables see Table 2

**Fig. SI2** The correlations between variables (measurements) taken from the radioulna. For a definition of variables see Table 2

**Fig. SI3** The correlations between variables (measurements) taken from the metacarpus. For a definition of variables see Table 2

**Fig. SI4** The correlations between variables (measurements) taken from the femur. For a definition of variables see Table 2

**Fig. SI5** The correlations between variables (measurements) taken from the tibia. For a definition of variables see Table 2

**Fig. SI6** The correlations between variables (measurements) taken from the metatarsus. For a definition of variables see Table 2

**Table S1** The selection of variables for variable reduction for whole bones in the pLDA models (Inclusion in pLDA) and the selection of variables for variable reduction for proximal and distal bone parts (Proximal/distal) for the limb bones

| **Element** | **Variable** | **Inclusion in pLDA** | **Proximal/distal** |
| --- | --- | --- | --- |
| Humerus | GL | No | No |
| Humerus | GLC | Yes | No |
| Humerus | GLl | No | No |
| Humerus | Bp | Yes | Proximal |
| Humerus | SD | Yes | No |
| Humerus | CD | No | No |
| Humerus | Bd | No | Distal |
| Humerus | BT | Yes | Distal |
| Humerus | HT | Yes | Distal |
| Humerus | DC | Yes | Proximal |
| Humerus | HTC | No | Distal |
| Humerus | Dp | Yes | Proximal |
| Humerus | Dd | Yes | Distal |
| Humerus | PL | No | No |
| Femur | GL | No | No |
| Femur | GLC | Yes | No |
| Femur | Bp | Yes | Proximal |
| Femur | DC | Yes | Proximal |
| Femur | SD | Yes | No |
| Femur | SDD | No | No |
| Femur | CD | No | No |
| Femur | Bd | Yes | Distal |
| Femur | Dd | Yes | Distal |
| Femur | BT | Yes | Distal |
| Femur | PL | No | No |
| Metacarpus | GL | Yes | No |
| Metacarpus | Bp | Yes | Proximal |
| Metacarpus | Dp | Yes | Proximal |
| Metacarpus | SD | Yes | No |
| Metacarpus | CD | No | No |
| Metacarpus | Bd | Yes | Distal |
| Metacarpus | BTm | No | Distal |
| Metacarpus | BTl | No | Distal |
| Metacarpus | DVm | No | Distal |
| Metacarpus | DVl | No | Distal |
| Metacarpus | BAp | No | Proximal |
| Metacarpus | BDF | Yes | Distal |
| Metacarpus | BA | Yes | Distal |
| Metacarpus | GCD | No | No |
| Metacarpus | GDD | No | No |
| Metacarpus | SDD | No | Distal |
| Metacarpus | PL | No | No |
| Metacarpus | DFp | No | Proximal |
| Metatarsus | GL | Yes | No |
| Metatarsus | Bp | Yes | Proximal |
| Metatarsus | Dp | Yes | Proximal |
| Metatarsus | SD | Yes | No |
| Metatarsus | CD | No | No |
| Metatarsus | Bd | Yes | Distal |
| Metatarsus | BTm | No | Distal |
| Metatarsus | BTl | No | Distal |
| Metatarsus | DVm | No | Distal |
| Metatarsus | DVl | No | Distal |
| Metatarsus | BAp | No | Proximal |
| Metatarsus | BDF | Yes | Distal |
| Metatarsus | BA | Yes | Distal |
| Metatarsus | GCD | No | No |
| Metatarsus | GDD | No | No |
| Metatarsus | SDD | No | Distal |
| Metatarsus | PL | No | No |
| Metatarsus | DFp | No | Proximal |
| Radioulna | GL | No | No |
| Radioulna | PL | Yes | No |
| Radioulna | Bp | Yes | Proximal |
| Radioulna | BFp | No | Proximal |
| Radioulna | CD | No | No |
| Radioulna | Bd | Yes | Distal |
| Radioulna | SD | Yes | No |
| Radioulna | SDD | No | No |
| Radioulna | Dd | Yes | Distal |
| Radioulna | Dp | Yes | Proximal |
| Radioulna | LO | Yes | Proximal |
| Radioulna | SDO | Yes | Proximal |
| Tibia | GL | No | No |
| Tibia | Ll | Yes | No |
| Tibia | SD | Yes | No |
| Tibia | SDD | No | No |
| Tibia | CD | No | No |
| Tibia | Bd | No | Distal |
| Tibia | Dd | Yes | Distal |
| Tibia | Dp | Yes | Proximal |
| Tibia | PL | No | No |
| Tibia | BFp | Yes | Proximal |
| Tibia | BFd | Yes | Distal |
