# Supplementary figures and images for: The osteometric identification of castrated reindeer (Rangifer tarandus) and the significance of castration in tracing human-animal relationships in the North
Source: Archaeol Anthropol Sci. 2022 Dec 9;15(1):3. doi: 10.1007/s12520-022-01696-y (PMC9734228; doi:10.1007/s12520-022-01696-y)

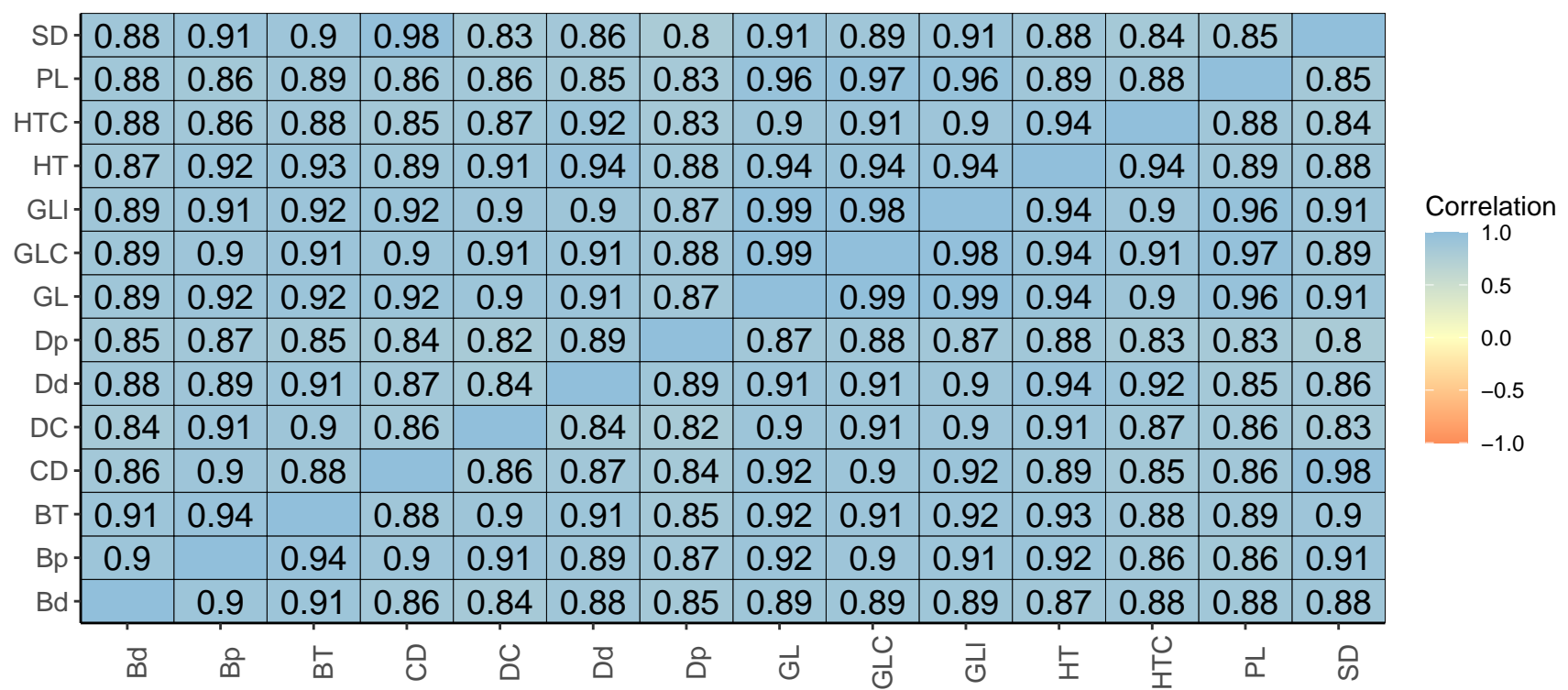

Supplement: Supplementary file 2 — Supplementary file2 (PDF 8 KB) [file 12520_2022_1696_MOESM2_ESM.pdf]

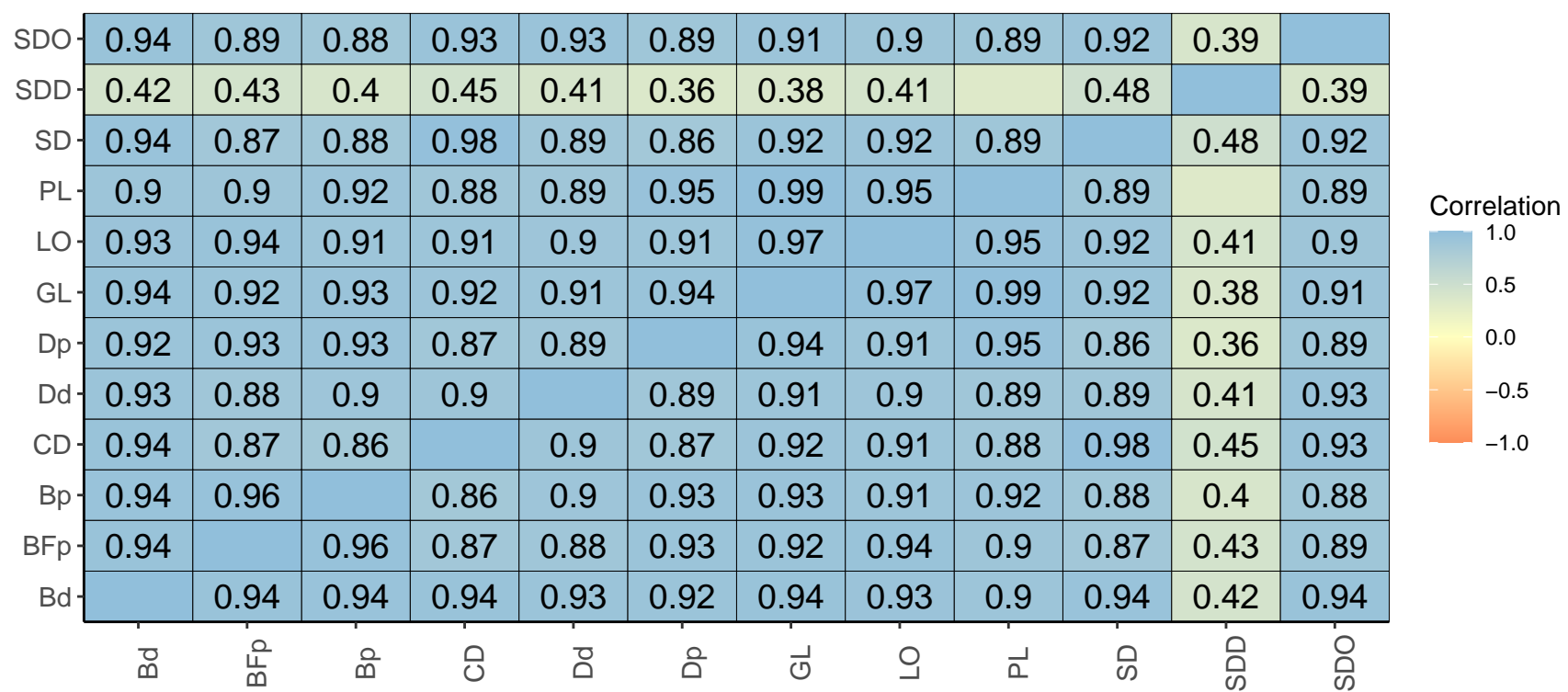

Supplement: Supplementary file 3 — Supplementary file3 (PDF 7 KB) [file 12520_2022_1696_MOESM3_ESM.pdf]

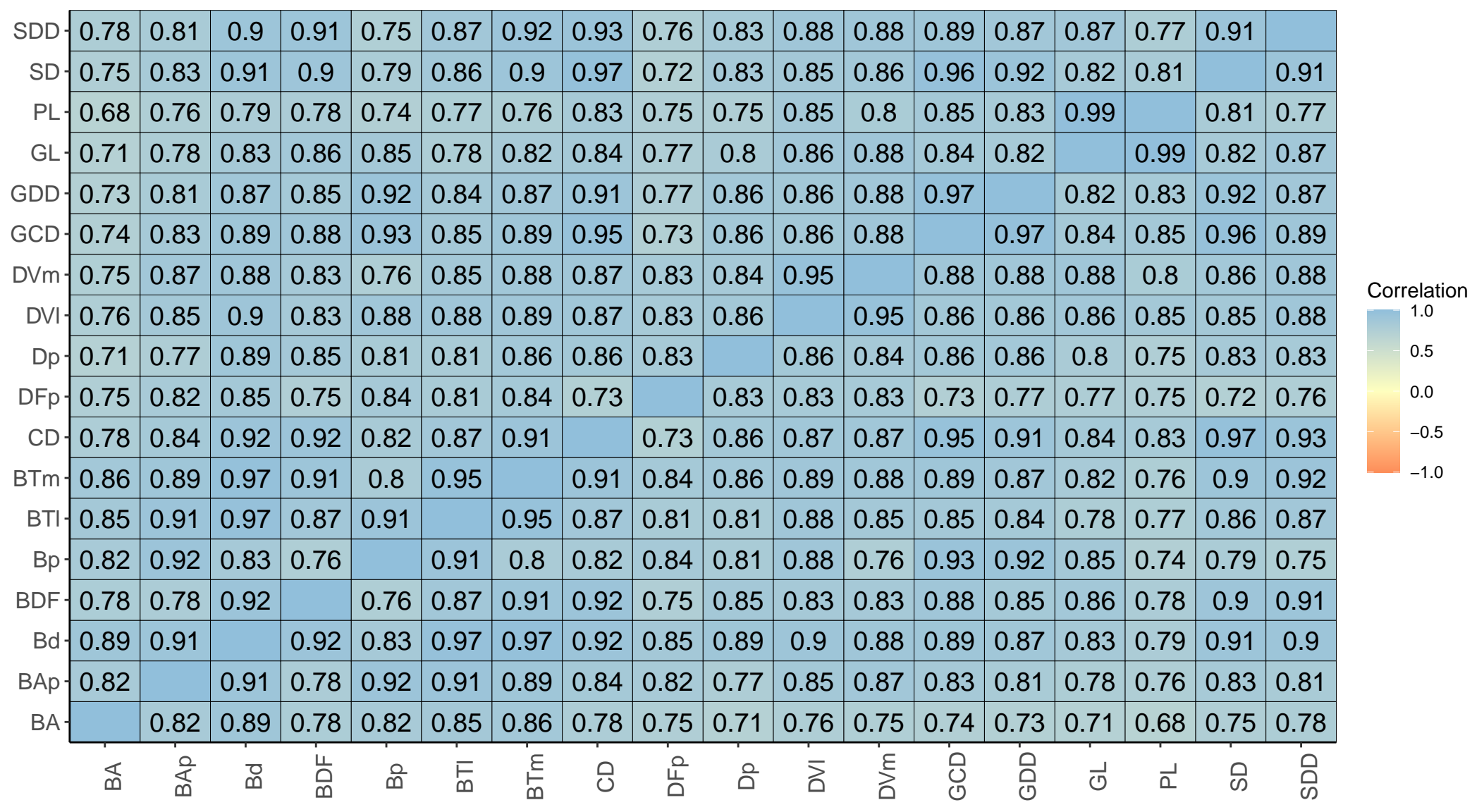

Supplement: Supplementary file 4 — Supplementary file4 (PDF 10 KB) [file 12520_2022_1696_MOESM4_ESM.pdf]

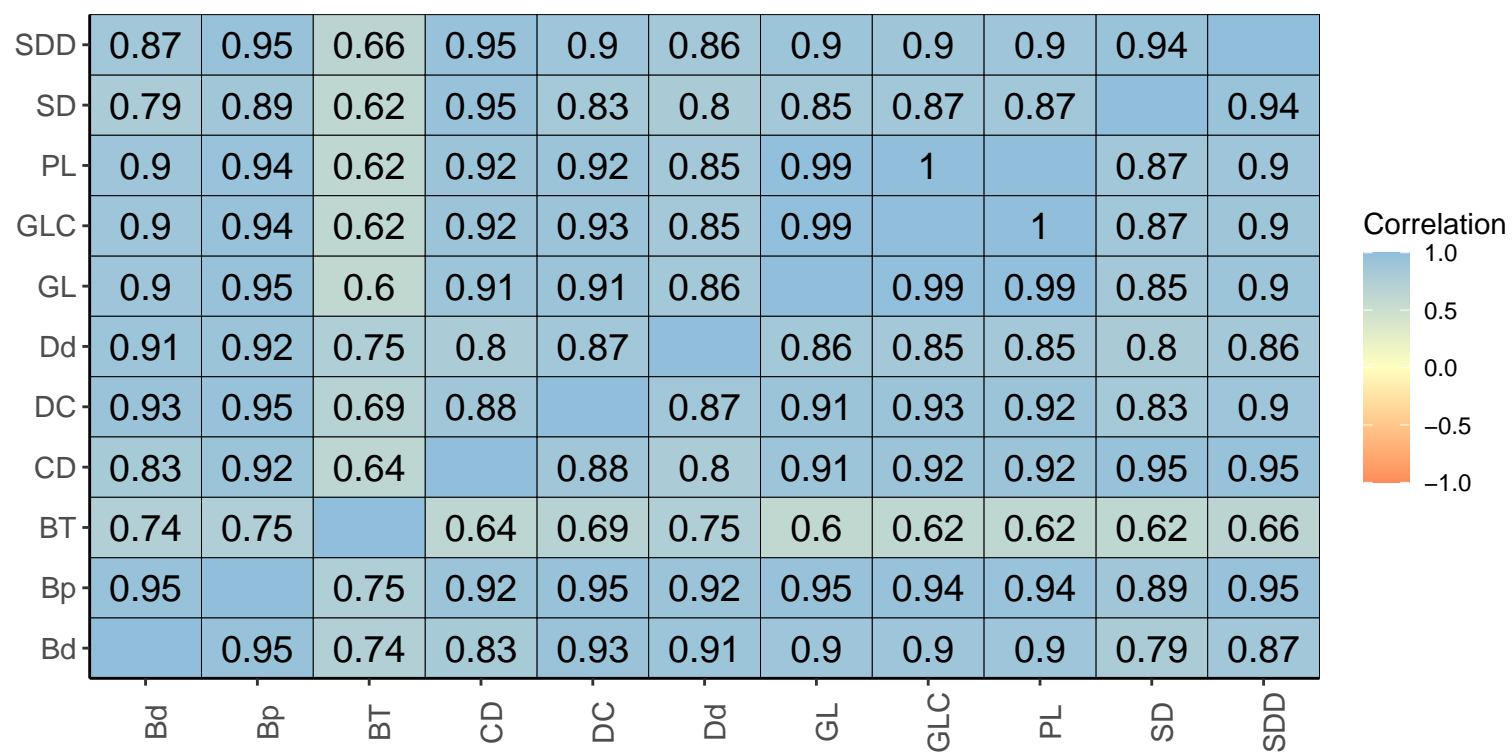

Supplement: Supplementary file 5 — Supplementary file5 (PDF 7 KB) [file 12520_2022_1696_MOESM5_ESM.pdf]

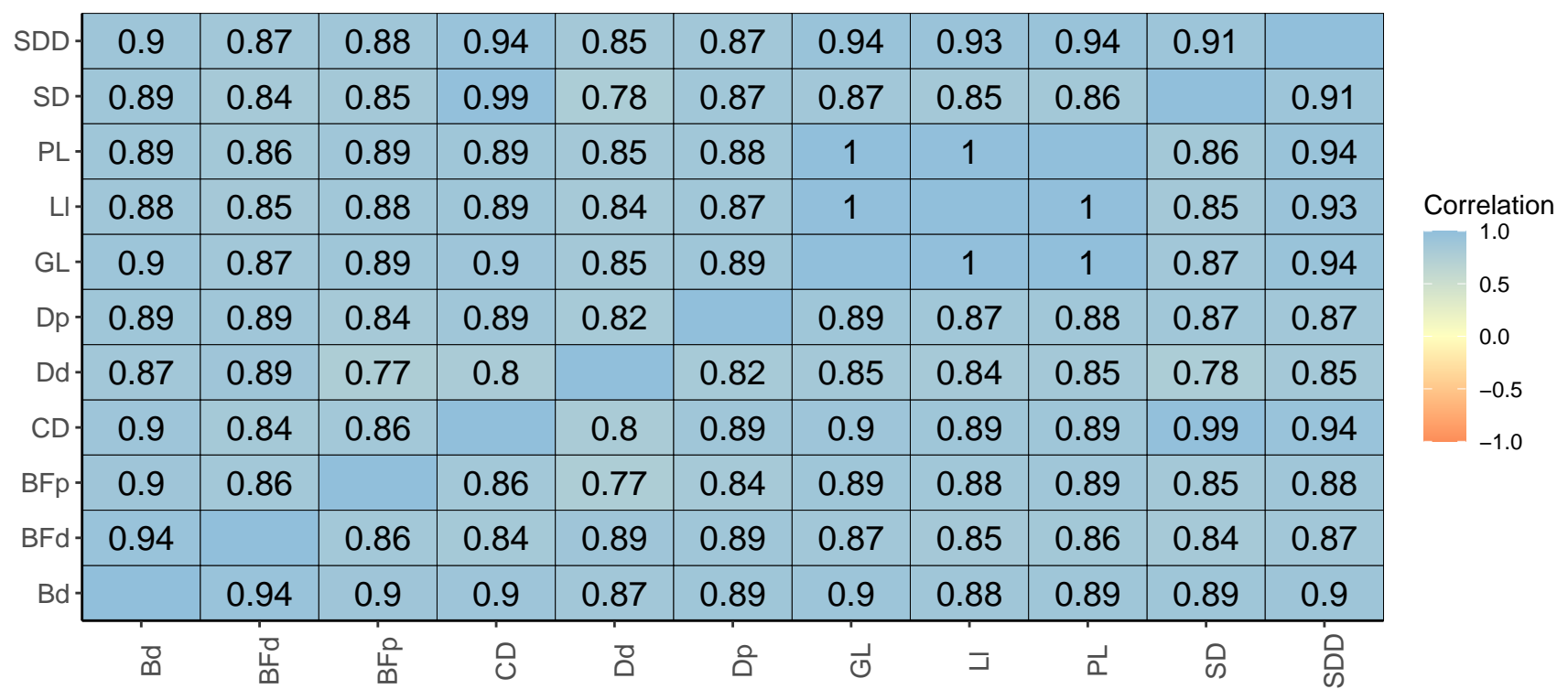

Supplement: Supplementary file 6 — Supplementary file6 (PDF 7 KB) [file 12520_2022_1696_MOESM6_ESM.pdf]

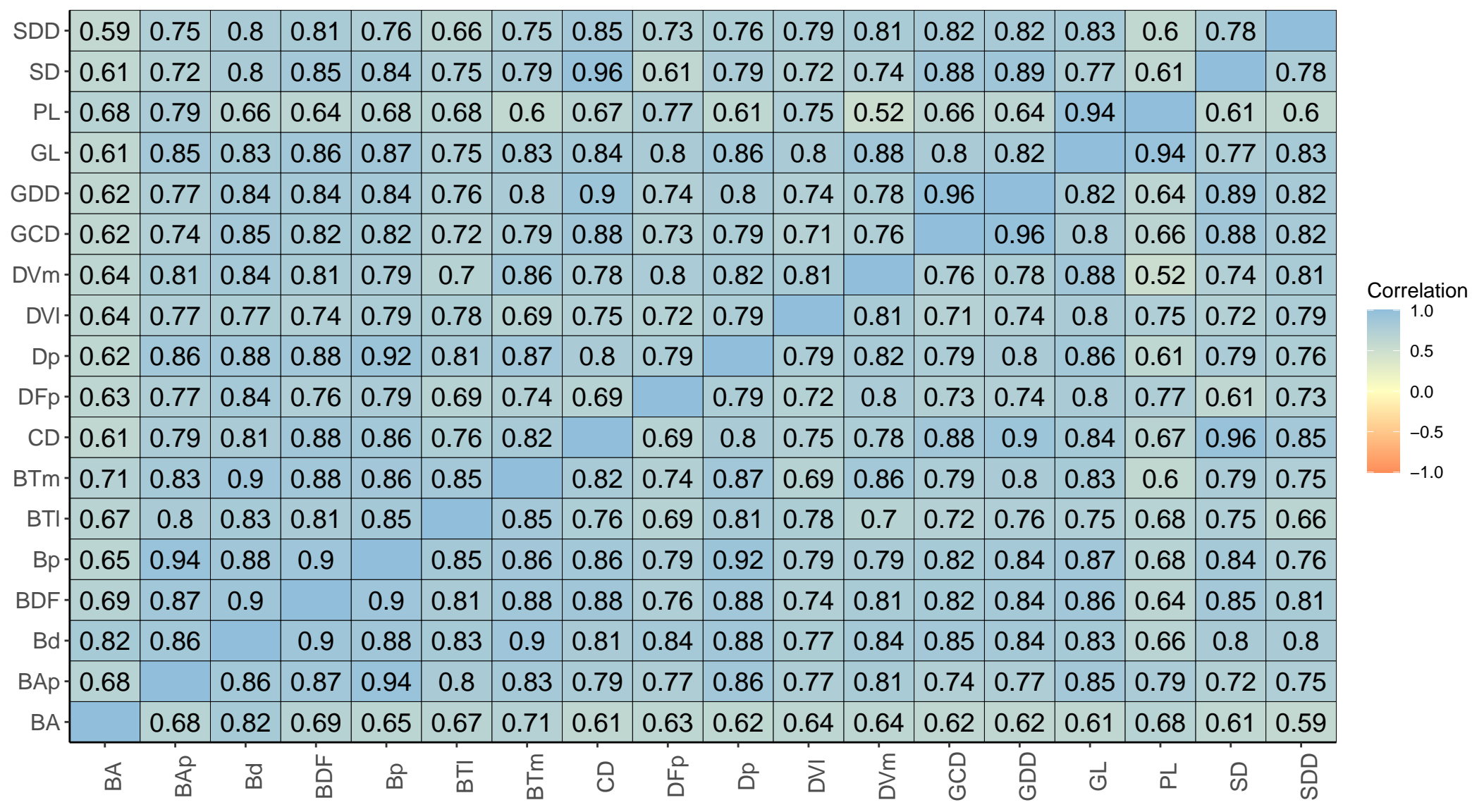

Supplement: Supplementary file 7 — Supplementary file7 (PDF 10 KB) [file 12520_2022_1696_MOESM7_ESM.pdf]

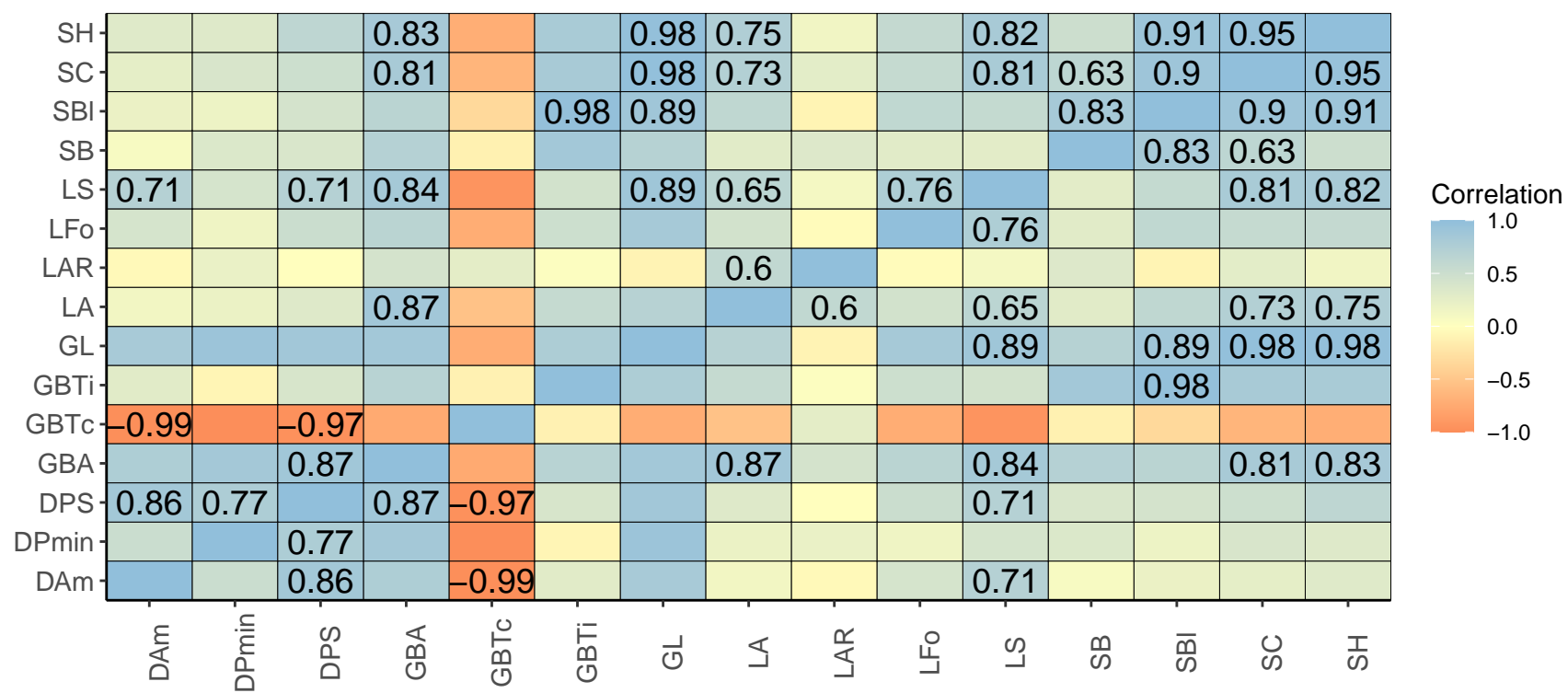

Supplement: Supplementary file 8 — Supplementary file8 (PDF 8 KB) [file 12520_2022_1696_MOESM8_ESM.pdf]
